# Clinical Epidemiology of Hypertension in Rural Thailand: A Nationwide Cross-Sectional Study

**DOI:** 10.5334/gh.1515

**Published:** 2025-12-31

**Authors:** Boonsub Sakboonyarat, Kamakshi Lakshminarayan, Ram Rangsin, Mathirut Mungthin, Kanlaya Jongcherdchootrakul, Jaturon Poovieng

**Affiliations:** 1 Division of Epidemiology and Community Health, School of Public Health, University of Minnesota, Minneapolis, MN 55454, USA; 2 Department of Military and Community Medicine, Phramongkutklao College of Medicine, Bangkok 10400, Thailand; 3 Department of Parasitology, Phramongkutklao College of Medicine, Bangkok 10400, Thailand; 4 Pulmonary and Critical Care Division, Department of Medicine, Phramongkutklao College of Medicine, Bangkok 10400, Thailand

**Keywords:** hypertension, blood pressure, cardiovascular disease, risk factors, rural Thailand

## Abstract

**Background:**

The clinical epidemiology of hypertension (HTN) in rural Thailand has not been fully reported. We describe factors associated with HTN control and cardiovascular (CV) outcomes in rural Thai communities.

**Methods:**

We conducted a cross-sectional study in Thai rural areas in 2024 using a multistage sampling scheme. Eligible participants included adults with HTN receiving care from 36 primary care units across four geographical regions. We used multilevel logistic regression modeling to examine factors associated with HTN control.

**Results:**

We included 1000 participants (68.3% women; mean age, 64.2 years). The HTN control rate was 63.9%, using a threshold of <140/90 mmHg for defining HTN control. When using optimal blood pressure thresholds (<130/80 mmHg for ages 20–64 years; <140/80 mmHg for ages ≥65 years), the HTN control rate was even lower at 47.8%. Factors associated with uncontrolled HTN included younger age, residence in the southern region, no school attendance, adding extra salt to food, low physical activity levels, and obesity. Prevalence of cardiovascular diseases (CVD) in people with HTN was as follows: stroke (10.3%), ischemic heart disease (1.4%), atrial fibrillation (1.2%), and left ventricular hypertrophy (6.0%). A high or very high 10-year CVD risk (i.e., > 20% risk over 10 years) was predicted in 7.9% of individuals with HTN: 12.7% in males and 5.0% in females. Elevated low-density lipoprotein (LDL) cholesterol (≥100 mg/dL) was present in 58.7% of participants; 51.5% had a body mass index of ≥25 kg/m^2^. Life’s Essential 8 CV health was categorized as poor, moderate, and high for 8.8%, 83.3%, and 7.8% of participants, respectively.

**Conclusions:**

We highlight a need for improving HTN control in rural Thailand and have identified sociodemographic, lifestyle, and metabolic factors that are associated with a lack of HTN control. Cardiovascular complications remain a significant concern for this population.

## Graphical abstract

**Figure:**
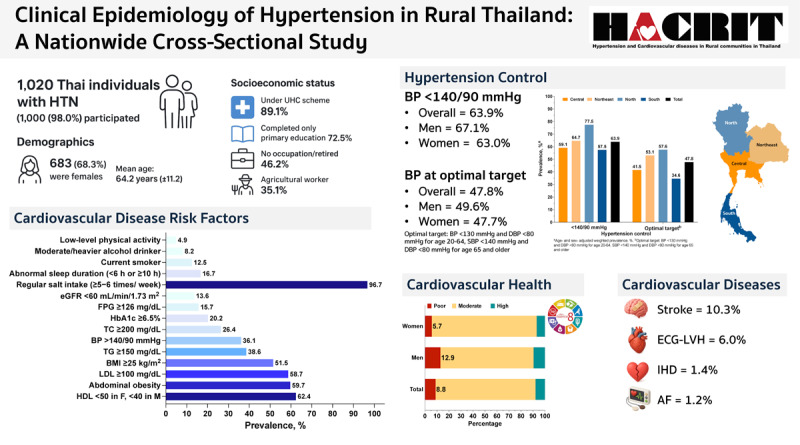


Clinical Epidemiology of Hypertension in Rural Thailand: A Nationwide Cross-Sectional Study (2024).

## Introduction

Hypertension (HTN) is a leading cause of premature death globally (1). Approximately 1.28 billion adults aged 30–79 years have HTN, with two-thirds living in low- and middle-income countries (1). Over the last three decades, Thailand has experienced a considerably higher burden of HTN than high-income nations (2). According to the National Health Examination Survey (NHES) (3), the prevalence of HTN in Thai adults increased from 23.1% to 26.8% in males and from 23.1% to 24.6% in females between NHES IV in 2009 and NHES VI in 2020 (3). The prevalence of HTN among urban Thai residents has remained high, ranging from 25.2 to 27.5%. Meanwhile, the prevalence in rural areas has increased from 21.1% in 2009 to 26.0% in 2020 (3).

The HTN component of the 2018 Thailand Diabetes Mellitus/Hypertension study examined 36,557 individuals with HTN who had received care for at least 12 months. The study reported an overall HTN control (<140/90 mmHg) rate of 66.6%, with 65.9% in males and 67.0% in females (4). HTN complication rates, including cardiovascular (CV) complications, were 4.3% (4). However, the study focused solely on patients receiving care at hospital-affiliated clinics. It lacked information on blood pressure (BP) control and HTN complications among those receiving care at primary care units (PCUs), especially in rural areas.

Measures of cardiovascular health (CVH) and cardiovascular disease (CVD) risk are useful for delivering preventive care (5). In 2022, the American Heart Association introduced “Life’s Essential 8,” highlighting lifestyle factors, including sleep duration, in CVH (5). The 2019 World Health Organization (WHO) CVD risk scores were established and then externally validated in the Thai population (c-index 0.72) (6). Our goal is to contribute to the data on CVH status and CVD risk in Thai populations with HTN.

Approximately 51% (7 million) of Thai adults with HTN reside in rural communities (3). However, the characteristics and clinical outcomes of these individuals have not been fully studied. We conducted the “HTN and CVD in Rural Communities in Thailand” (HACRIT) study, nationwide in geographically representative rural areas of Thailand, to evaluate the HTN control rates, factors associated with HTN control, CV risk factors, CVH, CVD, and predicted 10-year risk of CVD among people with HTN receiving continuous care in rural communities.

## Methods

### Study design and participants

The nationwide cross-sectional study was conducted in rural Thailand from June 26 to October 18, 2024. We included people with HTN aged ≥20 years who had received continuous care for at least 12 months at the PCU in a rural community. Individuals with HTN were identified based on the International Classification of Diseases, Tenth Revision (ICD-10) I10 diagnosis code (7).

We used a multistage sampling approach and invited eligible individuals to participate in the study. Stage 1 involved probability proportional to size (PPS) systematic sampling of 14 provinces across the four geographical regions (Figure S1). In Stage 2, we performed PPS systematic sampling of 18 districts (excluding the provincial capital district). Stage 3 consisted of simple random sampling to select two PCUs located outside a designated municipal area in each district, totaling 36 PCUs. Stage 4 involved eligible sampling; we performed a systematic random sampling of people with HTN from the eligible participants at each PCU.

At least 248 participants per region were needed to ensure good geographical representation of the HTN control rates (4). A total of 1,020 individuals with HTN across 36 PCUs were invited to participate in the study (Table S1). We explained the study details to the invited people, allowing them to voluntarily participate. All participants provided written consent. The study was reviewed and approved by the Institutional Review Board of the University of Minnesota (STUDY00020627) and the Institutional Review Board of the Royal Thai Army Medical Department in Thailand (S013h/66).

### Data collection

The data collection consisted of three components: (i) a paper-based questionnaire with face-to-face interviews, (ii) medical record review, and (iii) physical examination and laboratory tests. The questionnaire included demographics, socioeconomic characteristics, comorbidities, medication adherence (8), psychological conditions (9,10,11), and lifestyle factors. Completed questionnaires were collected, stored in sealed envelopes, and sent to the data management unit (DMU) in Bangkok, Thailand. Medical records were reviewed by trained registered nurses at the PCU. Data on the duration of HTN treatment, medication use, comorbidities, and history of CVD by ICD-10 codes were extracted, recorded in paper-based case report forms (CRFs), and sent to the DMU in sealed envelopes. Physical examinations were conducted at PCUs by well-trained staff.

Before the investigation, standardized training for the investigators was implemented. Participants’ BP was measured by one of the investigators (B.S.) using an automated oscillometric BP monitor (OMRON, HEM-7120, Kyoto, Japan) in adherence to the 2019 Thai guidelines for the treatment of HTN (12). Four measurements with one-minute intervals were carried out to allow for acclimation (13). Trained technicians performed anthropometric measurements, including height, body weight, waist circumference (14), and hip circumference (15). A trained staff member recorded a 12-lead electrocardiogram (ECG) using a 12-channel ECG machine (Model: MAC 2000 ECG, GE HealthCare, India). Clinical laboratory testing included hemoglobin A1c (HbA1c), fasting plasma glucose (FPG), lipid profiles (total cholesterol [TC], triglyceride [TG], low-density lipoprotein [LDL]-cholesterol, and high-density lipoprotein [HDL]-cholesterol), and serum creatinine. Study participants were asked to fast for at least 8 hours before the tests (16). Further details of data collection are provided in Supplemental Methods.

### Outcome variables

#### Antihypertensive medications use

We classified antihypertensive medications into five categories: (i) angiotensin-converting enzyme inhibitors (ACEIs) or angiotensin receptor blockers (ARBs); (ii) calcium channel blockers (CCBs); (iii) beta-blockers (BBs); (iv) diuretics; and (v) others (4). The number of antihypertensive medications used was categorized as no medication use, single therapy, dual therapy, polytherapy, and single-pill combination. Among participants who were prescribed antihypertensive medication, medication adherence was assessed on a scale of 9–36 based on the Hill-Bone Medication Adherence Scale (8,17).

#### HTN control

We used the average of the last three BP readings and defined HTN control following the 2019 Thai HTN guidelines (12) as a systolic BP (SBP) <140 mmHg and a diastolic BP (DBP) <90 mmHg. In addition, we defined optimal HTN control as SBP <130 mmHg and DBP <80 mmHg for individuals aged 20–64 years and SBP <140 mmHg and DBP <80 mmHg for those aged ≥65 years (12).

#### Cardiovascular risk factors

CV risk factors, including metabolic and lifestyle risk factors, are described in detail in Supplemental Methods. Metabolic risk factors included uncontrolled HTN, obesity, abdominal obesity, high waist-hip ratio, high TC, high TG, high LDL cholesterol, low HDL cholesterol, dyslipidemia (DLP), high FPG, high HbA1C, diabetes, and chronic kidney disease (CKD). Lifestyle risk factors included current smokers, current moderate/heavier drinkers (18), low level of physical activity (PA) (19,20), regular salt intake (21), and abnormal sleep duration (5).

#### CVH metrics score

We used Life’s Essential 8 CVH metrics, which include diet, PA, nicotine exposure, sleep duration, body mass index (BMI), blood lipids, blood glucose, and BP, to assess CVH (5). Details on these metrics are provided in Table S2. Based on a 0–100-point scale, scores of <50 signify poor CVH, those of 50–79 indicate moderate CVH, and those of ≥80 reflect high CVH (5).

#### CVDs

CVD, including stroke, ischemic heart disease (IHD), atrial fibrillation (AF), and ECG-left ventricular hypertrophy (ECG-LVH), are described in detail in Supplemental Methods. Briefly, we defined stroke based on the information from the questionnaire and medical record review (ICD-10 codes: I60–I64). We defined IHD using one of three sources: a questionnaire, a review of medical records (ICD-10 codes I20–I22 and I25 or a history of coronary revascularization), or findings from a 12-lead ECG indicating a prior myocardial infarction (22). We defined AF using one of two sources: medical record review, as the ICD-10 code I48, or findings from a 12-lead ECG indicating ECG-AF (23). We defined ECG-LVH based on one of three ECG criteria: the Peguero–Lo Presti criteria, Cornell voltage index, and Sokolow–Lyon criteria (24).

#### Predicted 10-year CVD risk

We used the 2019 WHO CVD risk score in participants aged 40–74 years without a history of CVD (stroke and IHD) to predict 10-year CVD risk. The laboratory-based model used age, sex, SBP, current smoking, diabetes (yes/no), and TC (mmol/L) to estimate the risk score (6). We converted TC from mg/dL to mmol/L by multiplying it by 0.02586 (25). The non-laboratory-based model used BMI instead of diabetes and TC to estimate the risk score. High or very high predicted 10-year CVD risk was defined as a risk score ≥20% (6).

### Statistical analysis

Data analyses were performed using the Stata Statistical Software: Release 17 (StataCorp, College Station, TX, USA). The analytic sample was weighted against the database for people with HTN receiving continuous care at each PCU in 2024. We used the svyset command for standard weighting procedures to construct sample weights considering the multistage sampling survey scheme.

Categorical variables are presented as percentages, and continuous variables are presented as mean and standard deviation. We estimated the HTN control rates, CV risk factors, CVH, CVD, and predicted 10-year risk of CVD. We also calculated the sex-adjusted, age-adjusted, and age- and sex-adjusted percentage or mean of the outcomes stratified by age group, sex, and geographical region, respectively.

We used multilevel regression analysis to identify factors associated with HTN control and estimated unbiased standard errors, with PCUs specified as a random effect. We used melogit and margin commands to obtain predicted probabilities and calculate the prevalence ratio. We estimated the adjusted prevalence ratio along with its corresponding 95% confidence interval using multivariable analysis. The final model included the following variables: sex, age, geographical region, health insurance scheme, marital status, education, occupation, duration of HTN, added extra salt before meals, PA, sleep duration, alcohol intake, smoking status, number of antihypertensive medications used, diabetes, DLP, CKD, BMI, and psychological test scales. A two-sided *p*-value of less than 0.05 was considered statistically significant. B.S. had full access to all the data in the study and takes responsibility for its integrity and the data analysis.

## Results

### Characteristics of study participants

Of the 1,020 Thai individuals with HTN who were invited to participate in the study, 1,000 (98.0%) participated. Table 1 presents the characteristics of the study participants. Among the respondents, 683 (68.3%) were females; the mean age was 64.2 years (±11.2). Most participants were under the universal health coverage (UHC) scheme (89.1%) and had completed only primary education (72.5%). Almost half of the participants (46.2%) reported having no occupation or being retired, and 35.1% were agricultural workers. Half of the participants (51.9%) had household incomes of less than 50,000 Thai Baht (~ $1,500) per year. The mean duration of HTN treatment was 8.3 (±5.3) years.

**Table 1:** Characteristics of study participants.


CHARACTERISTICS	MEN	WOMEN	TOTAL

	*n* = 317	*n* = 683	1000

	*n* (%)	*n* (%)	*n* (%)

**Sex distribution, %**	31.7	68.3	

**Age, years**			

20–29	2 (0.6)	1 (0.2)	3 (0.3)

30–39	7 (2.2)	15 (2.2)	22 (2.2)

40–49	20 (6.3)	51 (7.5)	71 (7.1)

50–59	65 (20.5)	158 (23.1)	223 (22.3)

60–69	115 (36.3)	236 (34.6)	351 (35.1)

70–79	87 (27.4)	164 (24.0)	251 (25.1)

≥80	21 (6.6)	58 (8.5)	79 (7.9)

mean (SD)	64.3 (11.0)	64.1 (11.2)	64.2 (11.2)

median (Q1–Q3)	65.0 (58.0–72.0)	65.0 (57.0–72.0)	65.0 (57.0–72.0)

**Geographical region**			

Central	78 (24.6)	176 (25.8)	254 (25.4)

Northeast	79 (24.9)	167 (24.5)	246 (24.6)

North	87 (27.4)	165 (24.2)	252 (25.2)

South	73 (23.0)	175 (25.6)	248 (24.8)

**Health insurance scheme**			

Universal health coverage	278 (87.7)	613 (89.8)	891 (89.1)

Civil servant medical benefits	24 (7.6)	53 (7.8)	77 (7.7)

Social security	9 (2.8)	10 (1.5)	19 (1.9)

Others	6 (1.9)	7 (1.0)	13 (1.3)

**Religion**			

Buddhism	292 (92.1)	636 (93.1)	928 (92.8)

Christian	19 (6.0)	35 (5.1)	54 (5.4)

Islam	6 (1.9)	11 (1.6)	17 (1.7)

Others	0 (0.0)	1 (0.2)	1 (0.1)

**Marital status**			

Married	249 (78.6)	425 (62.2)	674 (67.4)

Widowed	33 (10.4)	192 (28.1)	225 (22.5)

Divorced/separated	15 (4.7)	36 (5.3)	51 (5.1)

Never married	20 (6.3)	30 (4.4)	50 (5.0)

**Educational attainment**			

Never attend	29 (9.2)	87 (12.7)	116 (11.6)

Nonformal	2 (0.6)	2 (0.3)	4 (0.4)

Grade 1–6	213 (67.2)	512 (75.0)	725 (72.5)

Grade 7–9	25 (7.9)	35 (5.1)	60 (6.0)

Grade 10–12	26 (8.2)	32 (4.7)	58 (5.8)

Vocational	13 (4.1)	6 (0.9)	19 (1.9)

Bachelor’s degree or higher	9 (2.8)	9 (1.3)	18 (1.8)

**Occupation**			

No occupation/retired	113 (35.7)	349 (51.1)	462 (46.2)

Agricultural workers	143 (45.1)	208 (30.5)	351 (35.1)

Seller	22 (6.9)	72 (10.5)	94 (9.4)

Homemaker	2 (0.6)	28 (4.1)	30 (3.0)

Service	7 (2.2)	8 (1.2)	15 (1.5)

Government officer	5 (1.6)	5 (0.7)	10 (1.0)

Mechanic	8 (2.5)	0 (0.0)	8 (0.8)

Professional	3 (1.0)	2 (0.3)	5 (0.5)

Priest	4 (1.3)	0 (0.0)	4 (0.4)

Others	10 (3.2)	11 (1.6)	21 (2.1)

**Household income, per year**			

Under 50,000 Thai Baht	142 (44.8)	377 (55.2)	519 (51.9)

50,000–99,999 Baht	78 (24.6)	143 (20.9)	221 (22.1)

100,000–149,999 Baht	41 (12.9)	70 (10.3)	111 (11.1)

150,000–199,999 Baht	13 (4.1)	21 (3.1)	34 (3.4)

200,000–249,999 Baht	13 (4.1)	27 (4.0)	40 (4.0)

250,000–299,999 Baht	3 (1.0)	3 (0.4)	6 (0.6)

300,000 Baht and over	14 (4.4)	30 (4.4)	44 (4.4)

No intention to respond	13 (4.1)	12 (1.8)	25 (2.5)

**Duration of hypertension treatment, years**			

1–3	72 (22.7)	130 (19.0)	202 (20.2)

4–6	62 (19.6)	122 (17.9)	184 (18.4)

7–9	86 (27.1)	172 (25.2)	258 (25.8)

≥10	97 (30.6)	259 (37.9)	356 (35.6)

mean (SD)	8.0 (5.7)	8.5 (5.1)	8.3 (5.3)

median (Q1–Q3)	8.0 (4.0–11.0)	8.0 (4.0–12.0)	8.0 (4.0–11.0)

**Systolic blood pressure, mmHg**			

Mean (SD)	134.5 (16.8)	135.0 (17.0)	134.8 (16.9)

Median (Q1–Q3)	132.3 (124.7–143.7)	133.3 (123.3–144.3)	133.0 (123.7–144.4)

**Diastolic blood pressure, mmHg**			

Mean (SD)	77.2 (10.3)	78.4 (10.5)	77.6 (10.4)

Median (Q1–Q3)	76.3 (69.7–84.0)	78.0 (71.0–84.7)	76.7 (70.2–84.3)

**Body mass index, kg/m** ^2^			

Mean (SD)	24.2 (4.7)	25.8 (5.1)	25.3 (5.1)

Median (Q1–Q3)	23.7 (21.1–26.6)	25.5 (22.3–28.5)	24.8 (21.7–28.0)


SD: standard deviation, Q1–Q3: interquartile range.

### Prescription information for antihypertensive medications and medication adherence

Table S3 summarizes the prescription patterns of antihypertensive medications and medication adherence scores. Notably, 8.3% of individuals with HTN were not prescribed any antihypertensive medication. Single therapy was prescribed to 49.9% of people with HTN, while dual therapy and polytherapy were prescribed to 31.3% and 10.5% of participants, respectively. ACEI/ARB was the most common single therapy for those aged 20–64 years (35.6% for those aged 20–44 years and 61.7% for those aged 45–64 years), and CCB was predominantly prescribed for individuals aged ≥65 years (60.9%). For dual therapy, ACEI/ARB+CCB was the most frequent combination across all age groups (91.2%, 76.6%, and 78.7%, respectively). In polytherapy, ACEI/ARB+CCB+BB was mainly prescribed for individuals aged 20–44 (36.7%) and 45–64 (56.2%) years, while those aged ≥65 years typically received ACEI/ARB+CCB+Diuretic (69.6%). Among those on dual or polytherapy, only 0.5% used single-pill combinations. Notably, there was no use of a mineralocorticoid receptor antagonist. The overall mean medication adherence score was 33.9, with that of males and females averaging 33.2 and 34.4, respectively. In addition, 75% of individuals aged 20–44 years fell into the lowest tertile (T1) for medication adherence.

### HTN control

**Figure 1:**
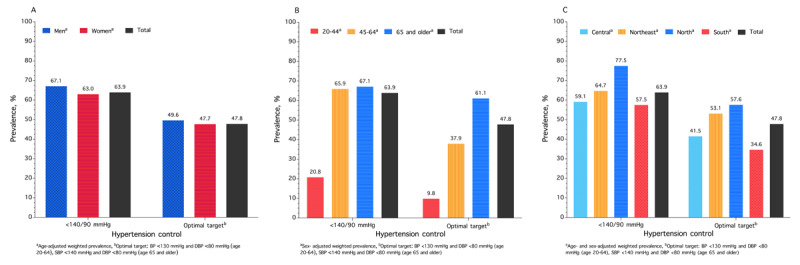
Prevalence of hypertension control among people with hypertension in rural Thailand in 2024, stratified by sex **(A)**, age **(B)**, and geographical region **(C)**.

Figure 1 presents the weighted prevalence of HTN control among people with HTN in rural areas of Thailand. The overall prevalence of HTN control (<140/90 mmHg) was 63.9%. The age-adjusted prevalence of HTN control was 67.1% for males and 63.0% for females. The sex-adjusted prevalence of HTN control was 20.8% for individuals aged 20–44 years, 65.9% for those aged 45–64 years, and 67.1% for those aged ≥65 years. The rates of HTN control varied by region, with the highest prevalence in the North (77.5%) and the lowest in the South (57.5%).

Next, we present statistics using an optimal threshold definition. The overall HTN control declined to 47.8%. The age-adjusted prevalence of HTN control was comparable between males (49.6%) and females (47.7%). The HTN control rates were significantly lower for younger individuals: 9.8% for those aged 20–44 years, 37.9% for those aged 45–64 years, and 61.1% for those aged ≥65 years. The HTN control rate in the North was 57.6%, while the South exhibited a lower HTN control rate of 34.6%. The distribution of observed BP in the study sample is presented in Tables S4 and S5.

### Factors associated with HTN control

Table S6 shows univariate analysis of factors associated with HTN control among Thai people with HTN in rural areas. Multivariate analyses are presented in Table 2. The HTN control rate was lower among younger individuals, those living in the Southern region, single individuals, and those who had never attended school. In addition, the prevalence of HTN control varied by occupation, household income, and health insurance scheme. Regarding lifestyle factors, individuals who usually added extra salt or salty sauces to their food (two or more times per week) and those who reported low levels of PA had lower rates of HTN control. Moreover, individuals with a BMI of ≥25.0 kg/m^2^ had a lower prevalence of HTN control than those with a BMI of ≤23.0 kg/m^2^.

**Table 2:** Multivariable analysis for factors associated with hypertension control among people with hypertension in rural Thailand.


FACTORS	HTN CONTROL (<140/90)		HTN CONTROL (OPTIMAL TARGET)*	
	
	ADJUSTED PR (95% CI)	*p*-VALUE	ADJUSTED PR (95% CI)	*p*-VALUE

**Sex**				

Women	Ref.		Ref.	

Men	1.05 (0.83–1.27)	0.663	1.00 (0.75–1.24)	0.970

**Age, years**				

20–44	0.64 (0.29–0.99)	0.046	0.27 (0.01–0.53)	<0.001

45–65	1.01 (0.77–1.26)	0.906	0.64 (0.57–0.71)	<0.001

≥65	Ref.		Ref.	

**Geographical region**				

North	Ref.		Ref.	

Central	0.87 (0.73–1.01)	0.067	0.84 (0.62–1.05)	0.143

Northeast	0.86 (0.72–0.99)	0.034	0.99 (0.82–1.17)	0.928

South	0.78 (0.66–0.91)	0.001	0.65 (0.57–0.74)	<0.001

**Health insurance scheme**				

Universal health coverage	Ref.		Ref.	

Civil servant medical benefits	0.92 (0.64–1.2)	0.555	0.78 (0.39–1.16)	0.256

Social security	1.28 (1.05–1.50)	0.016	1.41 (0.99–1.83)	0.058

Others	1.15 (0.79–1.50)	0.411	0.88 (0.49–1.27)	0.553

**Marital status**				

Married	Ref.		Ref.	

Widowed/divorced	0.94 (0.83–1.04)	0.253	0.78 (0.61–0.95)	0.010

Never married	0.61 (0.23–0.99)	0.049	0.86 (0.59–1.13)	0.302

**Educational attainment**				

Ever education attainment	Ref.		Ref.	

Never attend	0.84 (0.73–0.95)	0.004	1.01 (0.85–1.16)	0.928

**Occupation**				

No occupation/retired	Ref.		Ref.	

Farming/agriculture	1.08 (0.93–1.24)	0.302	1.01 (0.85–1.17)	0.897

Others	0.88 (0.75–1.01)	0.068	0.89 (0.71–1.07)	0.250

**Household income per year, Thai Baht**				

Under 50,000	Ref.		Ref.	

50,000–99,999	0.77 (0.61–0.93)	0.005	0.80 (0.61–0.99)	0.036

100,000–149,999	0.85 (0.66–1.05)	0.141	0.75 (0.56–0.94)	0.008

150,000 and over	0.78 (0.60–0.97)	0.026	0.93 (0.66–1.19)	0.576

No intention to respond	1.12 (0.80–1.44)	0.477	1.53 (1.01–2.04)	0.044

**Duration of hypertension treatment, years**				

1–3	Ref.		Ref.	

4–6	1.10 (0.95–1.25)	0.210	1.12 (0.76–1.48)	0.519

7–9	1.09 (0.96–1.22)	0.158	1.36 (0.96–1.75)	0.078

≥10	0.92 (0.83–1.00)	0.063	1.01 (0.71–1.31)	0.953

**Add extra salt or salty sauce**				

1 time per week and lower	Ref.		Ref.	

2–4 times per week and higher	0.62 (0.37–0.87)	0.003	0.47 (0.18–0.76)	<0.001

**Physical activity level**				

High	Ref.		Ref.	

Moderate	0.98 (0.87–1.09)	0.753	0.88 (0.73–1.03)	0.127

Low	0.29 (0.01–0.75)	0.002	0.29 (0.01–0.68)	<0.001

**Sleep duration, hours**				

<8	Ref.		Ref.	

≥8	1.08 (0.85–1.30)	0.512	1.12 (0.75–1.49)	0.524

**Smoking status**				

Never smoker	Ref.		Ref.	

Former smoker	1.08 (0.90–1.26)	0.407	1.14 (0.90–1.39)	0.253

Current smoker	1.03 (0.72–1.35)	0.839	1.00 (0.66–1.35)	0.984

**Alcohol use**				

Lifetime abstainer	Ref.		Ref.	

Former drinker	0.93 (0.73–1.12)	0.461	0.90 (0.72–1.08)	0.257

Current drinker	0.93 (0.76–1.11)	0.459	0.78 (0.50–1.05)	0.106

**Antihypertensive medication use**				

No medication use	0.76 (0.45–1.07)	0.239	0.76 (0.41–1.12)	0.191

Single therapy	Ref.		Ref.	

Dual therapy	1.13 (0.99–1.26)	0.083	1.39 (1.12–1.66)	0.004

Poly therapy	1.20 (1.01–1.39)	0.037	1.43 (1.16–1.70)	0.002

**Type 2 diabetes**				

No	Ref.		Ref.	

Yes	0.92 (0.75–1.08)	0.321	0.85 (0.61–1.08)	0.202

**Hyperlipidemia**				

No	Ref.		Ref.	

Yes	0.89 (0.54–1.24)	0.533	1.30 (0.52–2.08)	0.451

**Chronic kidney disease**				

No	Ref.		Ref.	

Yes	0.92 (0.70–1.14)	0.467	0.86 (0.58–1.14)	0.323

**Body mass index, kg/m** ^2^				

<23.0	Ref.		Ref.	

23.0–<25.0	0.93 (0.77–1.08)	0.338	0.88 (0.71–1.05)	0.170

≥25	0.86 (0.74–0.99)	0.028	0.85 (0.70–0.99)	0.035

**Psychological stress**				

Low	Ref.		Ref.	

Moderate	1.02 (0.92–1.11)	0.722	0.92 (0.77–1.07)	0.260

High	0.93 (0.17–2.03)	0.896	0.97 (0.01–2.30)	0.966

**Depression**				

Non-minimal	Ref.		Ref.	

Mild to moderately severe	1.19 (0.98–1.39)	0.079	1.39 (0.96–1.82)	0.074

Moderate to severe	0.99 (0.39–1.58)	0.965	1.32 (0.67–1.98)	0.335

**General anxiety**				

Minimal	Ref.		Ref.	

Mild to severe	1.36 (1.05–1.66)	0.023	1.10 (0.84–1.37)	0.449


*Systolic blood pressure<130 mmHg and diastolic blood pressure <80 mmHg for those aged 20–64 years, systolic blood pressure <140 mmHg and diastolic blood pressure <80 mmHg for those aged 65 years and older. HTN: hypertension, PR: prevalence ratio, CI: confidence interval.

### Cardiovascular risk factors

The prevalence of CV risk factors is shown in Table 3. Obesity prevalence was 51.5%: 41.7% among males and 56.1% among females. The prevalence of abdominal obesity was 59.7%: 35.7% in males and 72.5% in females. Notably, higher prevalence rates were observed in younger individuals. The highest prevalence was found in the Central region (59.6% for obesity and 75.1% for abdominal obesity). The overall prevalence of high LDL cholesterol was 58.7%, 56.2% in males and 59.9% in females. Individuals aged <65 years were more likely to have elevated LDL cholesterol levels. The Northeast region had the highest prevalence of elevated LDL cholesterol (65.0%). The overall prevalence of diabetes was 33.2%, with 24.6% in males and 37.7% in females. The Central region showed an exceptionally high prevalence of diabetes at 47.1%, followed by the Northeast at 40.5%, while the North and South reported lower rates of 18.7% and 19.4%, respectively. The overall prevalence of CKD was 13.6%, with 4.7% in males and 19.1% in females. The northeast region reported a substantially higher CKD prevalence at 22.6%.

**Table 3:** Prevalence of cardiovascular risk factors among people with hypertension in rural Thailand in 2024, stratified by sex, age, and geographical region.


CARDIOVASCULAR RISK FACTORS	AGE-ADJUSTED SEX-SPECIFIC WEIGHTED %		SEX-ADJUSTED AGE-SPECIFIC WEIGHTED %			AGE- AND SEX-ADJUSTED GEOGRAPHICAL REGION-SPECIFIC WEIGHTED %				TOTAL
		
	MEN	WOMEN	20–44	45–64	≥65	CENTRAL	NORTHEAST	NORTH	SOUTH	

**Metabolic risk factors**										

Dyslipidemia^§^	92.5	98.7^†^	100	95.6	97.4	97.1	98.0	94.8	95.1	96.5

High waist to hip ratio (≥0.90 in men and ≥0.85 in women)	56.0	77.0^‡^	90.2	73.3	65.9	82.9	65.9	71.7	73.3	69.7

Low HDL cholesterol (<40 mg/dL in men, < 50 mg/dL in women)	69.7	58.2	67.2	64.5	59.0*^c^	55.9^‡f^	54.3^†g, †h^	67.1	79.9	62.4

Abdominal obesity (≥90 cm in men, ≥80 cm in women)	35.7	72.5^†^	85.2	71.4	49.3^‡c^	75.1^‡e^	56.1	53.0^†i^	68.1	59.7

High LDL cholesterol (≥100 mg/dL)	56.2	59.9	66.0	69.4	48.7^†c^	56.4	65.0*^g, †h^	52.4	50.9	58.7

Obesity (BMI ≥25 kg/m^2^)	41.7	56.1*	89.0*^a^	62.2^†b^	38.7	59.6^‡e^	52.5*^g^	35.5^‡i^	54.0	51.5

High triglyceride (≥150 mg/dL)	40.0	37.8	36.0	32.8	43.7	44.0*^f^	36.3	52.4^†i^	28.7	38.6

Uncontrolled hypertension (>140/90 mmHg)	32.9	37.0	79.2^†a^	34.1^†b^	32.9	40.9^†e^	35.3*^g^	22.5^‡i^	42.5	36.1

Diabetes^||^	24.6	37.7*	54.1	35.8	29.6	47.1^†e, ‡f^	40.5	18.7	19.4	33.2

High total cholesterol (≥200 mg/dL)	25.7	26.3	55.6	20.9	27.8	24.9	26.5	25.5	24.6	26.4

Hyperglycemia (hemoglobin A1c ≥6.5%)	16.3	21.8	52.8	19.0^‡b^	17.8	30.5*^e, ‡f^	23.8	13.1	9.0	20.2

Hyperglycemia (fasting plasma glucose ≥126 mg/dL)	12.8	16.7	50.5*^a^	15.5*^b^	12.1	27.3^†e, ‡f^	15.7	10.0	10.5	15.7

Chronic kidney disease^#^	4.7	19.1*	1.2	4.9*^b^	24.2^‡c^	9.6*d,*^f^	22.6^†g, ‡h^	8.8	5.4	13.6

**Lifestyle risk factors**										

Regular salt intake (at least 5–6 times per week)	96.5	96.8	97.7	97	96.3	94.3^†e, †f^	95.1^†g, †h^	99.8	99.8	96.7

Abnormal sleep duration (<6 hours or ≥10 hours)	11.4	19.9*	7.4	15.7	19.5	14.8*^f^	17.3	8.9^‡i^	25.2	16.7

Current smoker	32.1	1.4^‡^	7.6	14.5	8.6	11.0	11.1	11.4	9.9	12.5

Moderate/heavier alcohol drinker (>3 drinks per week)	16.5	3.5^†^	6.2	11.2	4.6	9.9	7.2	10.2	7.4	8.2

Low-level physical activity	7.5	3.5*	0^‡a^	2.4^‡b^	7.4*^c^	3.3	6.3	1.2	4.2	4.9


BMI: body mass index, LDL: low-density lipoprotein. HDL: high-density lipoprotein. *p < 0.05, ^†^*p* < 0.01, ^‡^*p* < 0.001, ^§^Dyslipidemia was defined according to ICD-10 code E78 or a history of lipid-lowering medication use, high TC, high TG, high LDL cholesterol, or low HDL cholesterol, ^||^Diabetes was defined based on ICD-10 code E11, a history of antihyperglycemic medication use, high FPG, or high HbA1C. ^#^Chronic kidney disease was defined as an estimated glomerular filtration rate <60 mL/min/1.73 m^2^ or receiving renal replacement therapy. ^a^20–44 vs. 45–64, ^b^20–44 vs. 65 and older, ^c^45–64 vs. 65 and older, ^d^Central vs. Northeast, ^e^Central vs. North, ^f^Central vs. South, ^g^Northeast vs. North, ^h^Northeast vs. South, ^i^North vs. South.

Regarding lifestyle risk factors, the overall prevalence of current smokers was 12.5%, with a significantly higher rate in males (32.1%) than in females (1.4%). The overall prevalence of moderate to heavy alcohol consumption was 8.2%, with a higher rate in males (16.5%) than in females (3.5%). Most people reported moderate to high levels of PA: overall 95.1%, 92.5% in males and 96.5% in females. Most people (96.7%) reported regular salt intake. These rates were comparable across geographical regions. Abnormal sleep duration affected 16.7% of participants, 11.4% of males and 19.9% of females.

### CVH

**Figure 2:**
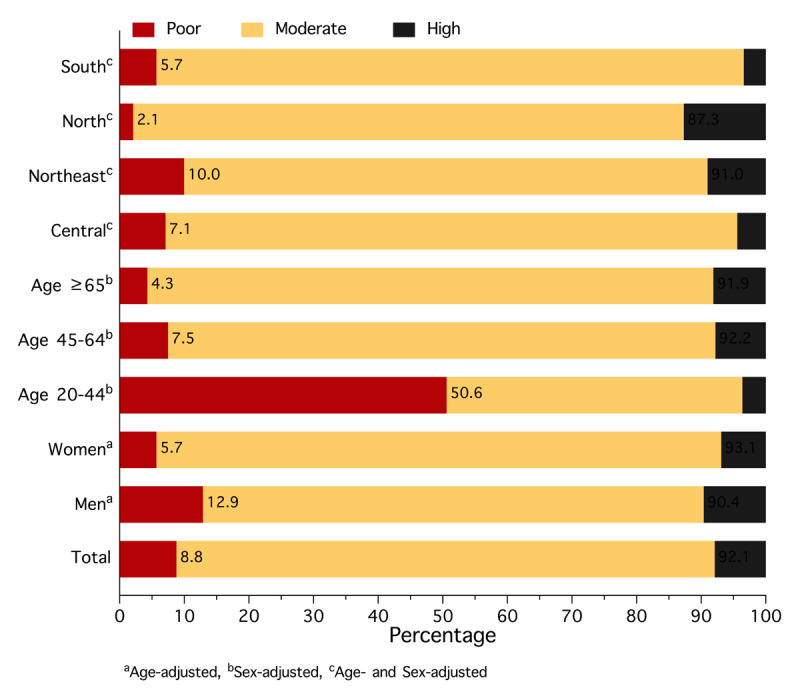
Cardiovascular health status (Life’s Essential 8) among people with hypertension in rural Thailand in 2024, stratified by sex, age, and geographical region.

Overall, the mean CVH score for the study population was 65.5, with scores of 64.6 for males and 66.2 for females (Table S7). Figure 2 presents CVH scores in three categories: 8.8% of individuals had poor CVH, 83.3% had moderate CVH, and 7.8% had high CVH. Poor CVH was recorded in 12.9% of males and 5.7% of females. Higher prevalence rates were observed in younger individuals, specifically those aged 20–44 years (50.6%). In addition, CVH varied across geographical regions; the poor CVH rate was highest in the Northeast (Table S8).

### CVDs

**Figure 3:**
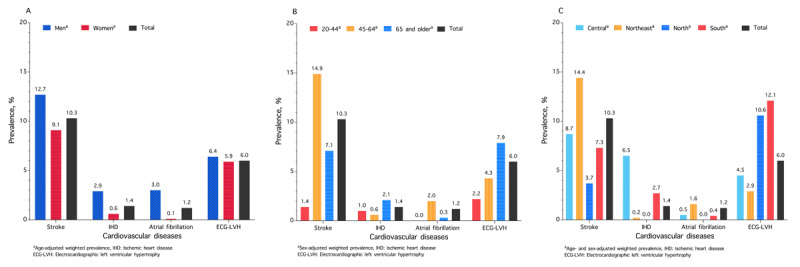
Prevalence of cardiovascular diseases among people with hypertension in rural Thailand in 2024, stratified by sex **(A)**, age **(B)**, and geographical region **(C)**.

Figure 3 shows the prevalence of CVD. Overall, stroke prevalence was 10.3% (12.7% in males, 9.1% in females), with regional variation: 14.4% in the northeast and 3.7% in the north. Overall, 81.7% of all strokes were ischemic, 11.7% were hemorrhagic, and 6.7% were unspecified subtypes. The prevalence of IHD was 1.4% overall (2.9% in males, 0.6% in females), peaking at 6.5% in the central region. AF prevalence was 1.2% (3.0% in males, 0.1% in females), while ECG-LVH was 6.0% overall (6.4% in males, 5.9% in females), ranging from 2.9% in the Northeast to 12.1% in the South. Prevalence rates for specific ECG-LVH criteria were 4.2% (Peguero–Lo Presti), 2.3% (Cornell voltage index), and 1.2% (Sokolow–Lyon). Details of CVD are presented in Tables S9 and S10.

### Predicted 10-year cardiovascular risk

**Figure 4:**
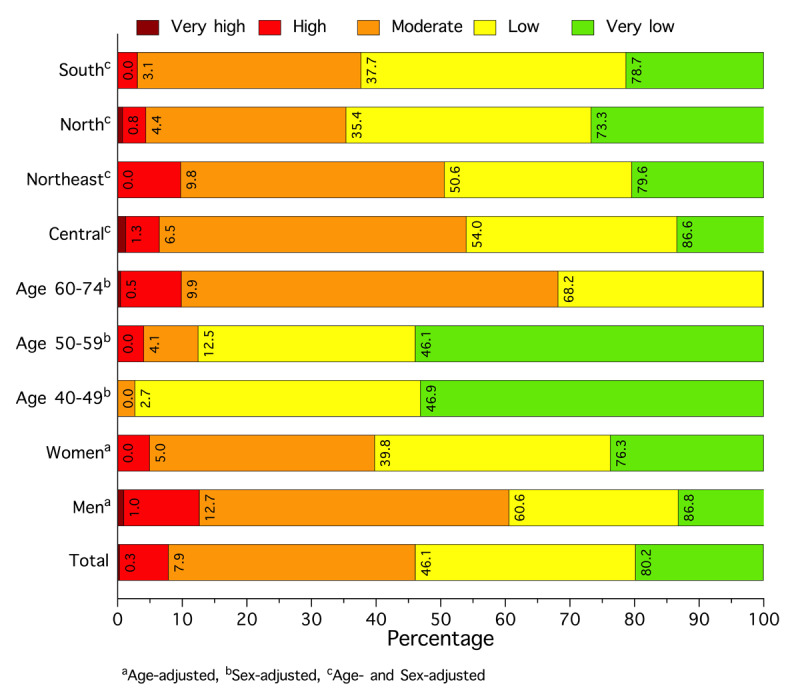
Distribution of predicted 10-year cardiovascular disease (CVD) risk using the World Health Organization laboratory-based risk chart among people with hypertension aged 40–74 without a history of CVD in rural Thailand.

To estimate the predicted 10-year CVD risk, we excluded 80 individuals with a history of IHD or stroke, as well as 182 individuals aged <40 or >74 years, leaving 738 individuals for risk prediction (Table S11). The overall mean laboratory-based prediction of 10-year CVD risk was 9.9%, with males at 11.7% and females at 9.0% (Table S12). Figure 4 illustrates the distribution of predicted 10-year CVD risk, stratified into five categories ranging from very low to very high. The prevalence of high or very high predicted 10-year CVD risk was 7.9% overall, with a higher rate in males (12.7%) than in females (5.0%) (Figure S2). Prevalence varied by geographical region, with the highest rates found in the Northeast (9.8%), followed by Central (6.3%), North (4.3%), and South (3.1%) regions. The predicted 10-year CVD risk calculated based on a non-laboratory-based score demonstrated a pattern similar to that of a laboratory-based score (Tables S12–S14).

## Discussion

The HACRIT study examined the clinical epidemiology of HTN in geographically representative rural areas of Thailand and found that, using a threshold of <140/90 mmHg, approximately two-thirds of the adults with HTN had controlled HTN. However, this rate was lower, at 47.8%, when a lower optimal threshold was used to define control. Demographics, lifestyle, and metabolic factors were associated with HTN control. Poor CVH was prevalent among younger individuals. CVDs were an important concern in this rural population. Hence, there is a need to improve both metabolic and lifestyle-related CV risk factors.

### Characteristics of people with HTN receiving care

The NHES VI (2020) in Thailand found that HTN prevalence among adults was comparable for males (26.8%) and females (24.6%) (3). The rates of undiagnosed HTN were 56.5% in males and 40.5% in females. Of those receiving treatment, 38.4% were males and 61.6% were females (26). In rural areas, one-third of people with HTN receiving care—35.2% across 36 PCUs (Table S18) and 31.7% in the HACRIT study—were males, which is lower than the proportions reported in both the NHES VI and the 2018 nationwide study (38.5%) (4). Our findings suggest that access to care for Thai males with HTN, particularly in rural areas, remains inadequate, potentially due to differences in health-seeking behavior between sexes (27). In Thailand, the PCU operates a community-based health screening program for adults aged ≥35 years (28), focusing on HTN, diabetes, and CV risk factors. Expanding this program to include adults under 35, specifically those aged 18 and older, could improve accessibility (29). In addition, setting separate coverage goals based on sex could facilitate the timely diagnosis and treatment of HTN.

### Treatment pattern

The HACRIT study found that half (49.9%) of the individuals with HTN were prescribed a single antihypertensive medication, while two-thirds (41.8%) required two or more. These findings differed from those of a 2018 study, which reported that 53.3% of adults with HTN in hospital clinics were on dual or polytherapy (4). The most common single therapy for those aged <65 years was ACEI/ARB, whereas CCB was mainly prescribed for those aged ≥65 years. For dual and polytherapy, ACEI/ARB+CCB and ACEI/ARB+CCB+Diuretic were primarily prescribed, aligning with Thai guidelines (12). Single-pill combinations may enhance medication adherence in individuals with HTN on dual or polytherapy (12,30), yet these combinations were only used by 0.5% of participants on dual or polytherapy. Implementing these single-pill combinations could improve HTN control and CVD prevention (31); however, the cost-effectiveness in rural areas requires further evaluation.

### HTN control rates

To our knowledge, the HACRIT study is the first to evaluate HTN control rates using regular and optimal thresholds. Overall, 63.9% of participants achieved HTN control, higher than the rate of 45.6% reported in a rural community study in the central region in 2018 (32) but lower than the rate of 66.6% reported in hospital clinics nationwide (4). We found that about two-thirds of individuals aged ≥45 years achieved HTN control, aligning with previous findings (4), while control rates for those aged 20–44 years in rural areas were substantially lower, at one-fifth and one-tenth for regular and optimal thresholds, respectively. We emphasize the need to improve HTN control among people with HTN in rural areas of Thailand, especially among younger individuals. Targeted measures should focus on reducing medication inertia through early antihypertensive medication initiation and lifestyle modifications. Based on our findings (Table S3), enhancing medication adherence is crucial for this group (33).

We note that the HACRIT study was ongoing in August 2024 when the 2024 Thai Guidelines for the treatment of HTN were initially released. These guidelines recommend an optimal target for HTN control of <130/80 mmHg, even for individuals aged ≥65 years (34). When applying this threshold, our HTN control rate is estimated to be lower, at 38.3%. However, although the guidelines were officially released in August 2024, free digital access was not provided until July 2025 (35). Therefore, it will be useful to re-evaluate HTN control rates with this threshold in the future, after the guidelines are fully implemented.

### Factors associated with HTN control and cardiovascular risk factors

Our study reveals significant regional disparities in HTN control rates, with only one-third of individuals in the Southern region achieving optimal control—far lower than that in other areas. These results remained consistent even after adjusting for lifestyle and metabolic conditions. This finding also aligns with that of a 2018 nationwide study (4); thus, there is a need to enhance the quality of HTN care in rural Southern areas. Future mixed-methods research using the socio-ecological model (36) should provide more comprehensive insights for developing targeted interventions for this population.

Existing evidence indicates that social determinants of health (SDOH) significantly influence HTN control (37). We observed that HTN control rates differ based on sociodemographic factors such as marital status, education, occupation, and health insurance scheme. Unmarried individuals typically have poorer control rates, possibly due to the lack of supportive health behaviors associated with marriage (38,39). Those without a formal education showed notably lower HTN control rates with the higher threshold, although no significant difference was observed in achieving optimal targets. Most participants were either unemployed or retired, or worked in agriculture, with both groups having similar HTN control rates. However, individuals in other occupations had lower rates of HTN control compared to those who were unemployed or retired. Disparities in HTN control were also observed across health insurance schemes, with those under UHC exhibiting lower control than those under social security, consistent with the findings of a previous nationwide study (4). Socioeconomic status in this population was not uniform and significantly associated with HTN control. Therefore, addressing SDOH is needed to improve HTN control in rural Thailand (37,40).

We found that lifestyle factors, such as high salt intake and low PA, were associated with lower HTN control rates. Those who added extra salt or salty sauce to meals at least twice a week had 38% and 53% lower HTN control rates (<140/90 mmHg and optimal target, respectively) compared with those who added them once a week or less often. Traditional Thai recipes often include salt and fish sauce, resulting in a high salt intake (41). Adding extra salt or sauces to meals can further increase sodium consumption. We recommend dietary modifications by eliminating extra salt in meals and reducing the use of salt and salty sauces (12,42).

Although over 95% of study participants reported moderate to high PA levels, those with low PA had significantly lower HTN control rates than those with high PA levels. Assessing PA levels in people with HTN when they visit the PCU may help identify those with low PA, allowing healthcare workers to encourage increased PA in this target group. This may facilitate HTN control and improve other metabolic risk factors (43,44).

Obesity significantly increases the risk of elevated BP and CVD (45,46), and our study showed that individuals with obesity had lower HTN control rates than those with normal weight. Half of the individuals with HTN in rural areas had obesity, higher than that among Thai adults in rural areas (40.8%) reported by the NHES VI (26). In addition to PA, we suggest a comprehensive weight management program (47) that includes health literacy promotion for healthy eating (48), daily self-weighing for awareness, and social support (49). It is essential to educate the population on the broader benefits of weight loss beyond HTN control, such as lowering the risk of CVD, for promoting behavioral change (50).

Our study found that approximately 60% of individuals with HTN had high LDL cholesterol, particularly the younger adults. Further exploration revealed that 36% of those with high LDL had not been prescribed lipid-lowering medication, and 13% had not undergone testing in the previous year. Addressing high LDL cholesterol and improving screening coverage for individuals with HTN in rural areas is necessary for mitigating atherosclerotic CVD risk.

### CVH

The HACRIT study is the first to assess CVH among individuals with HTN in Thailand. Notably, we observed moderate scores for most CVH metrics, with particularly poor ratings for BP and diet, highlighting the need for better HTN management and healthier eating habits. Half of the participants aged <45 years displayed poor CVH, which was linked to high obesity rates and uncontrolled HTN, hyperglycemia, and hyperlipidemia. We highlight the feasibility of using Life’s Essential 8 metrics for CVH assessment in this population and suggest developing computer-based tools for improved monitoring and personalized health advice (51).

### CVDs and predicted CVD risk

We found that one-tenth of people with HTN in rural Thailand experienced a stroke, with the highest prevalence in the Northeast (14.4%). Ischemic strokes were more common than hemorrhagic strokes. IHD was reported in 1.4% of participants, with a peak of 6.5% in the Central region. Among individuals aged 40–74 years without a history of CVD, a high or very high predicted 10-year CVD risk was more prevalent in the Northeast and Central regions. These findings highlight disparities in CVD and predicted 10-year CVD risks across various geographical regions. This information is crucial for guiding health policy and creating targeted key performance indicators to reduce regional CVD risk.

The Thai CV risk score, available for individuals aged 30–70 (52,53), has shown evidence of overestimation and weak discrimination in external validation studies (54,55). Consequently, we used the 2019 WHO CVD risk score for the Southeast Asian population in the HACRIT study. This WHO CVD risk score is properly validated in the Thai population (6) and can be used to facilitate regional comparisons. We found a strong correlation between the laboratory- and non-laboratory-based scores (Table S13), highlighting the utility of the non-laboratory score in limited-resource and rural settings (56). We also provided the predicted 10-year CVD risk based on the Thai CV risk score for reference (Tables S15–S17).

We found an overall AF prevalence of 1.2% (0.2% from medical records and 1.0% from 12-lead ECGs), lower than that reported in a prior nationwide study (2.8%) (4). However, the AF prevalence we reported may be underestimated, as only 10.2% of individuals with HTN in rural areas underwent 12-lead ECG screening, compared to approximately 17% in hospital clinics (57). Although all participants in our study underwent a 12-lead ECGs, it is possible that the test did not detect paroxysmal AF in this population.

In rural areas, 6.0% of individuals with HTN had ECG-LVH, and we found regional variations in prevalence (2.9% in the Northeast vs. 12.1% in the South). This disparity may stem from lower HTN control rates in the South. Both International and Thai guidelines recommend 12-lead ECG screenings for all individuals with HTN (12,42). Research shows that ECG screening of all individuals with HTN can yield significant benefits and could significantly reduce CV deaths more effectively than hemoccult screening for colon cancer death (58). Increasing 12-lead ECG screening in rural areas may improve management and reduce the risk of future complications.

### Limitations and strengths

We acknowledge the following limitations. First, we acknowledge that the study has a cross-sectional design and hence is not intended to establish a causal relationship between HTN control and antecedent risk factors for poor control. Second, the absence of data on distance to PCUs prevented analysis by rurality; we nevertheless found that HTN control prevalence varied notably by sociodemographic characteristics. Third, because the target study population is only rural residents, there is no direct urban comparator. Fourth, although a multistage sampling design was used to ensure national rural representativeness, clustering may limit diversity within clusters. Fifth, our use of a 12-lead ECG to detect LVH is not optimal. While it is not a gold standard, we used this since it was an inexpensive and reasonable screening method for individuals with HTN. We used three criteria: the Peguero–Lo Presti criteria, which have the highest sensitivity (62%), the Cornell voltage index (92% specificity), and the Sokolow–Lyon criteria, which offer high specificity (98%) (59). Finally, our study likely underestimated AF prevalence since we again ascertained this using a 12-lead ECG. It was not feasible to use Holter monitors and Zio patch for this large population, given our resources.

Despite the limitations, our study has several strengths. The HACRIT study is the first to assess HTN control rates among individuals receiving continuous care in rural Thailand across multiple geographical regions. It was conducted in 36 rural communities from 14 provinces, making our findings representative of HTN control in rural areas nationwide. We conducted standard BP measurements directly, using consistent methods and trained staff to minimize variability. This distinguishes the HACRIT from prior studies relying on medical records (4,46). In addition, we conducted a 12-lead ECG for all participants, enhancing the accuracy of estimating the prevalence of ECG abnormalities among individuals with HTN in rural areas.

## Conclusion

We highlight an opportunity to improve HTN control rates among Thai individuals with HTN in rural areas. Socioeconomic status, lifestyle choices, and metabolic conditions influence HTN control. CVDs remain a significant concern for this population. There is potential to improve metabolic and lifestyle-related CV risk factors.

## Data Accessibility Statement

Due to ethical or legal restrictions, the data supporting this study are not publicly available.

## Additional File

The additional file for this article can be found as follows:

10.5334/gh.1515.s1 Supplemental Materials. Supplementary Methods – Tables S1 to S18; Figures S1 and S2; References for Supplementary Methods.
