# Supplementary material for: Clinical Epidemiology of Hypertension in Rural Thailand: A Nationwide Cross-Sectional Study
Source: Glob Heart. 2025 Dec 31;20(1):116. doi: 10.5334/gh.1515 (PMC12758107; doi:10.5334/gh.1515)
Supplement: Supplemental Materials. — Supplementary Methods – Tables S1 to S18; Figures S1 and S2; References for Supplementary Methods. [file gh-20-1-1515-s1.pdf]

SUPPLEMENTARY MATERIALS

Clinical Epidemiology of Hypertension in Rural Thailand: A Nationwide Cross-sectional Study

Boonsub Sakboonyarat, MD, MPH[1,2]*, Kamakshi Lakshminarayan, MD, PhD[1], Ram Rangsin, MD, MPH, DrPH[2], Mathirut Mungthin, MD, MS, PhD[3], Kanlaya Jongcherdchootrakul, MD, MPH, PhD[2], Jaturon Poovieng, MD[4]

[1] Division of Epidemiology and Community Health, School of Public Health, University of Minnesota, Minneapolis, MN 55454, USA

[2] Department of Military and Community Medicine, Phramongkutklao College of Medicine, Bangkok 10400, Thailand

[3] Department of Parasitology, Phramongkutklao College of Medicine, Bangkok 10400, Thailand

[4] Pulmonary and Critical Care Division, Department of Medicine, Phramongkutklao College of Medicine, Bangkok 10400, Thailand

***Corresponding author:**

Boonsub Sakboonyarat, MD, MPH

Department of Military and Community Medicine, Phramongkutklao College of Medicine, Bangkok 10400, Thailand

Telephone: +66+85-9545955

E-mail: boonsub1991@pcm.ac.th

Table of Contents

	Page
**Supplementary Methods**	4
**Supplementary Figure S1.** Sampled provinces	4
**Supplementary Table S1.** Number of sampled participants from stage 4 sampling (presented by province)	5
**Supplementary Table S2.** Metrics for measurement and quantitative assessment of cardiovascular health	12
**Supplementary Table S3.** Uses of antihypertensive medications for people with hypertension in rural Thailand	13
**Supplementary Table S4.** Distribution of observed blood pressure of people with hypertension in rural Thailand, stratified by sex and age group	14
**Supplementary Table S5.** Distribution of observed blood pressure of people with hypertension in rural Thailand, stratified by geographical region	15
**Supplementary Table S6.** Univariable analysis for factors associated with hypertension control among people with hypertension in rural Thailand	16
**Supplementary Table S7.** Cardiovascular health score among people with hypertension in rural Thailand, stratified by sex and age group	18
**Supplementary Table S8.** Cardiovascular health score among people with hypertension in rural Thailand, stratified by geographical region	19
**Supplementary Table S9.** Prevalence of cardiovascular diseases among people with hypertension in rural Thailand, stratified by sex and age group	20
**Supplementary Table S10**. Prevalence of cardiovascular diseasess among people with hypertension in rural Thailand, stratified by geographical region	21
**Supplementary Table S11.** Characteristics of people with hypertension aged 40-74 without a history of cardiovascular disease in rural Thailand	22
**Supplementary Table S12.** Predicted 10-year cardiovascular disease risk by World Health Organization cardiovascular disease risk chart people with hypertension aged 40-74 without a history of cardiovascular disease in rural Thailand	23
**Supplementary Table S13.** Pearson's correlation coefficient of the predicted 10-year CVD risk using the non-laboratory-based and laboratory-based 2019 World Health Organization CVD risk score	23
**Supplementary Table S14.** Prevalence of high or very high predicted 10-year cardiovascular disease risk among people with hypertension aged 40-74 without a history of cardiovascular disease in rural Thailand (2019 World Health Organization CVD risk score)	24

Page

Supplementary Table S15. Predicted 10-year cardiovascular disease risk by the Thai cardiovascular risk score for people with hypertension aged 30-70 without a history of cardiovascular disease in rural Thailand 25

Supplementary Table S16. Pearson's correlation coefficient of the predicted 10-year CVD risk using the non-laboratory-based and laboratory-based Thai cardiovascular risk score 25

Supplementary Table S17. Prevalence of high or very high predicted 10-year cardiovascular disease risk among people with hypertension aged 30-70 without a history of cardiovascular disease in rural Thailand (the Thai cardiovascular risk score) 26

Supplementary Table S18. Sex distribution of people with hypertension receiving care at 36 primary care units in rural Thailand 27

Supplementary Figure S2. Prevalence of high or very high predicted 10-year cardiovascular disease risk among people with hypertension aged 40-74 without a history of cardiovascular disease in rural Thailand 28

References for Supplementary Methods 29

Supplementary Methods

Sample size calculation

The primary aim of this study was to assess the prevalence of hypertension (HTN) control among Thai people with HTN who receive continuous care at primary care units (PCUs) in rural communities. A previous study in 2018 involving 36,557 Thai people with HTN receiving continuous care for at least 12 months at hospital clinics nationwide, reported that the prevalence of HTN control was 66.6%[1]. To estimate the prevalence of HTN control in each geographical region, we calculated the sample size of study participants for each geographical region separately. We used the following formula to estimate the infinite population proportion: $\frac{z^2 p(1-p)}{d^2} \times \frac{1}{Response\ rate}$. With alpha (α) = 0.05, error (d) = 0.066 (10% of prevalence), and an 80% response rate, the sample size was at least 248 individuals for each geographical region.

Sampling design

Thailand comprises 77 provinces divided into four geographical regions: 26 in the Central region, 20 in the Northeast, 17 in the North, and 14 in the South. We employed a multistage sampling approach and invited eligible individuals to participate in the study. Stage 1 involved probability-proportional-to-size (PPS) systematic sampling of 14 provinces across the four geographical regions (**Figure S1**). In Stage 2, we performed PPS systematic sampling of 18 districts (excluding the provincial capital district). Stage 3 used simple random sampling to select 2 PCUs outside a designated municipal area in each district, for a total of 36 PCUs. Stage 4 involved eligible sampling; we performed systematic random sampling of eligible participants with HTN from each PCU. The total of invited study participants was 1,020.

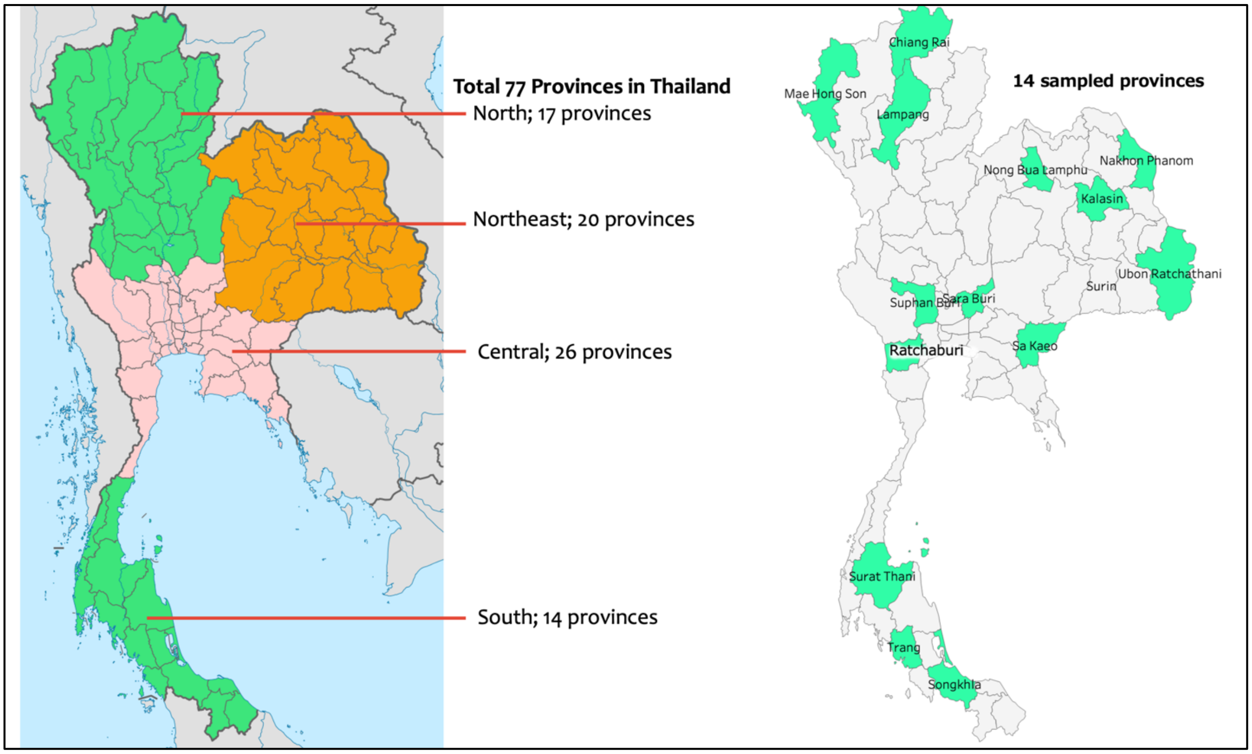

Supplementary Figure S1. Sampled provinces

Stage 1. Primary sampling selection (14 provinces)

The sampling unit was a province.

The sampling frame was the list of provinces in each geographical region.

The sampling method was simple random sampling.

Stage 2. Secondary sampling selection (18 Districts)

The sampling unit was a district.

The sampling frame was the list of districts in each province (excluding the provincial capital district).

The sampling method was probability-proportional-to-size systematic sampling.

Stage 3. Tertiary sampling selection (36 PCUs)

The sampling unit was a PCU.

The sampling frame was the list of PCUs outside the municipality (rural area) in each district.

The sampling method was simple random sampling (2 PCUs from each district).

Stage 4. Eligible sampling selection

The sampling unit was Thai people with HTN aged 20 years and older receiving care for at least 12 months at a PCU in a rural community.

The sampling frame was the list of people with HTN in each PCU. People with a hypertension diagnosis (ICD-10: I10) receiving care at PCU before June 1, 2023, appeared on the list.

The sampling method was systematic sampling in each PCU.

The total number of sampled participants from stage 4 sampling is presented in Table **S1**.

Supplementary Table S1. Number of sampled participants from stage 4 sampling (presented by province)

Sampled provinces	No. sampled districts	No. sampled PCUs	Total N from each PCU	Total N
**North**				
Chiang Rai	2	4	32	128
Lampang	1	2	32	64
Mae Hong Son	1	2	32	64
**Total**	**4**	**8**		**256**
**Northeast**				
Ubon Ratchathani	2	4	21	84
Kalasin	2	4	21	84
Nakhon Phanom	1	2	21	42
Nong Bua Lamphu	1	2	21	42
**Total**	**6**	**12**		**254**
**Central**				
Sa Kaeo	1	2	32	64
Ratchaburi	1	2	32	64
Suphan Buri	1	2	32	64
Saraburi	1	2	32	64
**Total**	**4**	**8**		**256**
**South**				
Surat Thani	2	4	32	128
Trang	1	2	32	32
Songkhla	1	2	32	32

Total	4	8	256
**TOTAL**	**18**	**36**	**1020**

Recruitment

We contacted the 36 targeted PCUs across Thailand's four geographical regions and provided them with information about the study. Subsequently, the investigators planned the data collection at the PCU. The list of eligible study participants was sampled from people with HTN receiving care at the PCU. Therefore, 1,020 sampled patients were invited to participate in the study. Participants were recruited from PCUs in rural communities. We explained the details of the study to eligible participants, and they would be free to decide whether to participate. Eligible participants who decided to participate in the study provided written consent. Data collection commenced only after obtaining written informed consent from the participants in accordance with the WMA Declaration of Helsinki Ethics Principles for medical research involving human subjects.

Data collection

A single team, comprising investigators and research assistants experienced in community-based research from Phramongkutklao College of Medicine (PCM), collected data at 36 PCUs across the country. The data collection consists of three components: a face-to-face interview, a review of medical records, and physical examination and laboratory tests.

The face-to-face interviews conducted as part of this study were designed to collect relevant demographic data from participants, including information on sex, age, occupation, household income, educational attainment, marital status, and health insurance scheme. Lifestyle factors such as tobacco use, alcohol consumption, physical activity, diet, and sleep duration would also be assessed. The interview included administering several standardized questionnaires, including the Hill-Bone Medication Adherence Scale (HB-MAS)[2,3], comprehensive health history, and psychological test scales, including the 10-item Perceived Stress Scale (PSS-10)[4], the 7-item General Anxiety Disorder Scale (GAD-7)[5]and the 9-item Patient Health Questionnaire (PHQ-9)[6]. Trained staff conducted the interviews, which lasted approximately 30 minutes each. Responses were recorded on a paper questionnaire, and each participant was assigned a unique identification number for tracking. Completed questionnaires were collected, sealed in envelopes, and sent to the data management unit (DMU) at PCM in Bangkok, Thailand.

For medical record review, trained registered nurses at the PCU carefully review participants' medical records alongside the study manual. The pertinent medical data, including the duration of HTN, antihypertensive medications, antihyperglycemic medications, lipid-lowering medications, antiplatelet/anticoagulant medications, comorbidities, and history of CVD by ICD-10, were extracted. A paper-based case report form (CRF) was utilized to record the information. Once the CRFs were completed, they were collected, stored in sealed envelopes, and sent to the DMU.

Each participant underwent a physical examination that measured their BP, took anthropometric measurements, and performed a 12-lead electrocardiography (ECG). BP was measured using an automated BP monitor (OMRON HEM-7120, Kyoto, Japan) by Dr. Sakboonyarat, in accordance with the 2019 Thai guidelines for the treatment of HTN[7]. The standard patient position for BP measurement was followed. Study participants were advised to

refrain from smoking and caffeine for at least 30 minutes before the measurement. They were instructed to remain seated for at least 5 minutes, with their arms supported at heart level and their feet firmly planted on the ground. During the measurement, participants were required to refrain from talking to prevent any interference. Four measurements were taken at one-minute intervals and were recorded. The average of the last three readings was then used as the blood pressure outcome to ensure precision in the measurements.

We gathered anthropometric measurements, including height, body weight, waist circumference, and hip circumference. Our well-trained technicians obtained these measurements. Height was measured with a wall-mounted ruler to 0.1 cm precision while participants were barefoot. Body weight and composition were assessed using a body composition monitor (OMRON model HBF-702, Kyoto, Japan). Waist and hip circumference were measured with an anthropometric tape measure, applied horizontally. Participants were instructed to stand upright, evenly distribute their weight between both feet, and breathe quietly. Waist circumference was measured at the level of the umbilicus for more consistency and accuracy [8]. Hip circumference was measured at the level of maximal protrusion of the gluteal muscles[9]. We used a partition to ensure privacy and confidence during the measurement.

The process of recording a standard 12-lead ECG was conducted by well-trained investigators using a 12-channel ECG machine (Model: MAC 2000 ECG, manufactured by GE Medical Systems Information Technologies Inc., India). Participants were instructed to lie down in a private partitioned room in a comfortable position, with their heads well-supported and their backs resting on the bed with pillows. To ensure accurate recordings, the inner aspects of the participants' wrists were positioned close to, but not touching, their waist**.** The investigator then carefully placed the limb leads on the participants' wrists and ankles. After that, the chest leads from V1 to V6 were applied. To ensure standardization, the calibration signal on the ECG machine was checked at a paper speed of 25 mm/s and an ECG size of 1 mV/10 mm deflection [10]. During the measurement, participants were asked to lie still and breathe normally. After that, the 12-lead ECG was recorded and accurately labeled with the identification number. A clinical preventive medicine physician (B.S.) interpreted the 12-lead ECG results, which were confirmed by an internist (J.P.).

Laboratory tests were conducted to assess various health indicators, including HbA1c, fasting plasma glucose, lipid profiles (total cholesterol (TC), triglycerides (TG), LDL-cholesterol, and HDL-cholesterol), and serum creatinine (Cr). Study participants were asked to fast for at least 8 hours before the tests[11]. The phlebotomy technicians drew blood samples and collected them in the tubes. The tubes were placed in an ice-water bath (4-8˚C) and dispatched to the central laboratory at PCM, Bangkok, Thailand.

Data management

The Research Unit for Military Medicine and the Office of Research and Development at the PCM in Thailand supported the study's data management. Each participant was assigned a unique study ID to ensure data safety and confidentiality. The link between the study ID and participant names was stored in secure locations accessible only to authorized personnel who needed to contact participants or provide care. All electronic storage was on secure servers and password-protected files. In accordance with institutional data practices, other records were archived for 10 years.

We used the REDCap system to manage the study data. Data storage was centralized

in a secure database maintained by the Office of Research Development at PCM, and access to this data was password-protected. In accordance with Good Clinical Practice principles, only de-identified data was analyzed. After data collection through the paper-based CRF, which involves conducting face-to-face interviews, reviewing medical records, and performing physical examinations, the collected data were sent to the data management unit at PCM for further processing. Dr. Sakboonyarat then transferred the data from the paper-based CRF onto the RedCap platform using a double data entry process.

Variables

Antihypertensive medications

According to antihypertensive medication use, we categorized them into five groups: (i) angiotensin-converting enzyme inhibitors (ACEI) or angiotensin receptor blockers (ARB); (ii) calcium channel blockers (CCB); (iii) beta-blockers (BB); (iv) diuretics; (v) others[12]. The information on the prescription from the latest visit was classified as no medication use, single therapy, dual therapy, polytherapy, and single-pill combination. Among participants who prescribed antihypertensive medication, the HB-MAS was used to assess medication adherence on a scale of 9−36, with a higher score indicating higher levels of medication adherence[2,3]. We considered HB-MAS as a discrete variable [2,3].

Hypertension control

Regarding HTN control, we measured BP in four readings and used the mean of the last three readings as the BP outcome. According to the 2019 Thai guidelines for the treatment of HTN, initial HTN control was defined as systolic BP (SBP) < 140 mmHg and diastolic BP (DBP) <90 mmHg. Optimal BP control was defined as SBP < 130 mmHg and DBP < 80 mmHg for individuals aged between 18–<65 years and SBP < 140 mmHg and DBP < 80 mmHg for those aged 65 and older[7]. The hypertensive crisis was defined as SBP ≥ 180 mmHg or DBP ≥ 110 mmHg[12].

Factors associated with hypertension control

We considered covariates that may be related to HTN control. These include demographic characteristics, lifestyle factors, the number of antihypertensive medications, comorbidities, psychological health, and anthropometric data. Demographic characteristics information was obtained through a face-to-face interview using a questionnaire that included age, sex, marital status, educational attainment, occupation, household income, geographical region, and health insurance schemes.

We considered dietary behavior, including dietary salt intake, based on the WHO STEPS instrument[13]. Dietary salt intake was assessed using a questionnaire that asked: "How often do you add salt or a salty sauce such as soy sauce or fish sauce to your food right before you eat it or as you are eating it?" Based on their response, we categorized their salt intake into two groups: at least 2−4 times per week and 1 time per week and lower[13].

The short version of the International Physical Activity Questionnaire (IPAQ) was used to assess physical activity over the last 7 days [14,15]. The IPAQ was reported as a continuous physical activity level in metabolic equivalent task (MET)-minutes per week or categorized as low, moderate, or high[14,15].

Sleep duration was assessed through the questionnaire by asking about the bedtime and wake-up time on weekends and weekdays. We then averaged the sleep duration between weekends and weekdays. The average sleep duration was categorized as <8 or≥8.

Smoking status was categorized into four groups, including never smoker, former smoker, someday smoker, and everyday smoker[16]. Current smokers were divided into "everyday smokers" or "someday smokers." An everyday smoker was an individual who had smoked at least 100 cigarettes in his or her lifetime and who now smokes every day. A Someday smoker was an individual who has smoked at least 100 cigarettes in his or her lifetime and smokes now but does not smoke every day. A former smoker was an individual who had smoked at least 100 cigarettes in his or her lifetime but had quit smoking at the time of the interview. A never-smoker was an individual who had never smoked or had smoked fewer than 100 cigarettes in his or her lifetime[16].

Alcohol use was categorized into four groups, including lifetime abstainer, former drinker, current infrequent/light drinker, and current moderate/heavier drinker[17]. A lifetime abstainer was an individual who had had fewer than 12 drinks in his or her lifetime. A former drinker was an individual who had consumed at least 12 drinks in their lifetime but no drinks in the past year. A current infrequent/light drinker was an individual who had had at least one drink in the past year but three drinks or fewer per week, on average, over the past year. A current moderate/heavier drinker was an individual who has had more than three drinks per week, on average, over the past year[17].

The number of antihypertensive medications used was extracted from the medical record and categorized into four groups, including no medication use (only lifestyle modification), monotherapy, dual therapy, and polytherapy[1].

The comorbidity that we considered consists of diabetes, hyperlipidemia, and chronic kidney disease (CKD). Diabetes was determined according to ICD-10: E11[18], a history of antihyperglycemic medication use or FPG ≥126 mg/dL, or HbA1C ≥6.5%[19]. Hyperlipidemia was determined according to ICD-10: E78[18], a history of lipid-lowering medication use, TC ≥200 mg/dL or LDL ≥100 mg/dL, TG ≥150 mg/dL, or HDL<40 in men and <50 in women. CKD was defined as an eGFR <60 mL/min/1.73 m^2 or having renal replacement therapy[1].

The PSS-10 was used to measure stress levels. The PSS-10 measures an individual's stress level on a scale of 0–40, with higher scores indicating higher perceived stress. The scores were categorized into three groups: 0−13, 14−26, and 27−40, which represent low, moderate, and high stress, respectively [4]. The GAD-7 scale was employed to assess anxiety levels. The GAD-7 measures anxiety severity on a scale of 0–21, with higher scores indicating higher anxiety levels. The scores were categorized into four groups: 0−4, 5−9, 10−14, and 15−21, representing minimal, mild, moderate, and severe anxiety, respectively [5]. The PHQ-9 was used to assess depression levels[5]. The PHQ-9 measures depression severity on a scale of 0−27, with higher scores indicating higher levels of depression. The scores were categorized into five groups: 0−4, 5−9, 10−14, 15−19, and 20−27, representing non-minimal, mild, moderate, moderately severe, and severe depression, respectively [6].

Anthropometric data also were considered, including BMI, waist circumference, and waist-to-hip ratio (WHR). BMI was calculated as body weight (kg) divided by height (m^2). BMI was categorized into five groups: 18.5−<23.0, <18.5, 23.0−<25.0, 25.0−<30.0, and ≥30, representing normal weight, underweight, overweight, obesity I, and obesity II, respectively[20].

Waist circumference was used to identify abdominal obesity, which was defined as a waist circumference ≥90 cm in men and ≥80 cm in women. WHR was calculated by dividing the waist circumference by the hip circumference and was cut off at ≥0.90 in men and ≥0.85 in women[21].

Cardiovascular risk factors

CV risk factors included uncontrolled HTN, obesity, abdominal obesity, high waist to hip ratio, high TC, high TG, high LDL, low HDL, hyperlipidemia, hyperglycemia, diabetes, CKD, current smoking, current moderate/heavier drinker, low level of physical activity, regular salt intake, and abnormal sleep duration.

Uncontrolled HTN was defined as SBP ≥140 mmHg or DBP ≥90 mmHg. Obesity was defined as a BMI ≥25 kg/m^2. Abdominal obesity was defined as a waist circumference ≥90 cm in men or ≥80 cm in women [20]. WHR was calculated by dividing the waist circumference by the hip circumference and cut off at ≥0.90 in men and ≥0.85 in women[21]. High TC was defined as TC ≥200 mg/dL, while High TG was defined as TG ≥150 mg/dL. High LDL cholesterol was defined as LDL ≥100 mg/dL, while low HDL cholesterol was defined as HDL <40 mg/dL in men or < 50 mg/dL in women [1]. We defined dyslipidemia according to ICD-10 code E78 or a history of lipid-lowering medication use, high TC, high TG, high LDL cholesterol, or low HDL cholesterol. Diabetes was determined according to ICD-10: E11, a history of antihyperglycemic medication use, FPG ≥126 mg/dL, or HbA1C ≥6.5%. CKD was defined as an eGFR <60 mL/min/1.73 m^2 or having renal replacement therapy[1].

We defined current smokers as individuals who have smoked 100 cigarettes in their lifetime and currently smoke cigarettes [16]. A current moderate/heavier drinker is an individual who has had more than three drinks per week, on average, over the past year[17]. A low level of physical activity was defined according to the IPAQ as the level that does not meet any of the criteria for either moderate or high levels of physical activity [14,15]. Regular salt intake was defined according to the WHO STEPS instrument, which is 1) adding salt or a salty sauce to food right before eating it or 2) salty seasoning or a salty sauce added in cooking or preparing foods in the household at least 5−6 times per week. Abnormal sleep duration was defined as short sleep duration (<6 hours) or long sleep duration (≥ 10 hours)[22].

Cardiovascular health

To assess cardiovascular health (CVH), we used Life's Essential 8 CVH metrics, which include diet, physical activity, nicotine exposure, sleep duration, BMI, blood lipids, blood glucose, and BP [22]. Each metric was assigned a score ranging from 0 to 100 points. The overall CVH score, ranging from 0 to 100 points, was calculated by averaging the scores obtained for each of the eight health measures. A score below 50 signifies poor CVH, 50−79 indicates moderate CVH, and 80 and above reflects high CVH. Table **S2** presents metrics for measuring and quantitatively assessing CVH.

Cardiovascular diseases

Cardiovascular diseases (CVD) included ischemic heart disease (IHD), stroke, atrial fibrillation (AF), and ECG-left ventricular hypertrophy (ECG-LVH). IHD was defined by one of three sources. The first source was a questionnaire that asked the subjects, "Have you ever been told by a doctor or other health worker that you have ischemic heart disease (heart

attack)?" The second was a history of IHD that appears in the medical records. IHD was defined as ICD-10 codes I20–I22 and I25, or a history of coronary revascularization documented in medical records. The third source was the 12-lead ECG findings, which indicated prior MI [23]. This was provided by the WHO definition as pathological Q waves [any Q wave in leads V2–V3 ≥0.02s—Minnesota code 1.2.1—or QS complex in leads V2 and V3—Minnesota code 1-2-7. Q-wave ≥0.03s and ≥0.1mV deep— Minnesota codes 1-1-1; 1-2-2—or QS complex in leads I, II, aVL, aVF, or V4–V6 in any two leads of a contiguous lead grouping I, aVL, V6: V4–V6: II, III, aVF (Minnesota codes 1-1-7; 1-3-6)] with or without symptoms [23].

Stroke was defined by one of two sources. The first source was a questionnaire that asked individuals whether they had ever been diagnosed with a stroke by a doctor or other healthcare provider and, if so, what type of stroke it was (ischemic, hemorrhagic, or unsure). The second source was the medical records that contain information on the patient's history of stroke. Stroke was defined based on the ICD-10 codes I60–I62 (cerebral hemorrhage), I63 (cerebral infarction), and I64 (stroke not identified as hemorrhage or infarction) in the medical record.

AF was defined by one of two sources. The first source was the patient's medical records, which contain information about their history of AF. AF was defined based on the ICD-10 code I48 recorded in the medical record. The second source was the findings from a 12-lead ECG, which indicated ECG-AF. The absence of P-waves, irregular undulations in the baseline, normal QRS duration but sometimes variable-shaped QRS complexes, and a totally irregular ventricular rate were considered ECG findings in AF, Minnesota code 8-3-1 and 8-3-3 [24].

ECG-LVH was diagnosed based on one of three ECG criteria: the Peguero-Lo Presti criteria, the Cornell voltage index, or the Sokolow-Lyon criteria. The Peguero-Lo Presti criteria: S_D+SV_4 ≥2.8 mV for men and S_D+SV_4 ≥2.3 mV for women. The Cornell voltage index: $RaVL+SV_3$ >2.8 mV for men and >2.0 mV for women. The Sokolow-Lyon criteria: SV_1+RV_5/RV_6 ≥3.5 mV[25].

Predicted 10-year cardiovascular disease risk

The predicted 10-year CVD risk was estimated by the 2019 WHO CVD risk score in participants aged 40-74 and without a history of CVD (IHD and stroke). The non-laboratory-based model used age (years), sex (men and women), SBP (mmHg), current smoking status (yes/no), and BMI (kg/m^2) to compute the risk [26]. The laboratory-based model used age, sex, SBP, current smoking, diabetes (yes/no), and TC (mmol/L) to estimate the risk [26]. We converted the TC from mg/dL to mmol/L by multiplying by 0.02586[27]. The 2019 WHO CVD risk scores were categorized as follows: <5% (very low), 5% to <10% (low), 10% to <20% (moderate), 20% to <30% (high), and ≥30% (very high) [26]. High or very high CVD risk was defined as a 10-year predicted CVD risk of ≥ 20% [26].

In addition, we calculated the predicted 10-year CVD risk using the Thai cardiovascular (CV) risk score in participants aged 30-70 years without a history of CVD (IHD or stroke)[28,29]. The non-laboratory-based model used age (years), sex (men and women), SBP (mmHg), current smoking status (yes/no), waist circumference (inches), and height (cm) to compute the risk. The laboratory-based model used age, sex, SBP, current smoking, diabetes (yes/no), and TC (mg/dL) to estimate the risk[28]. The Thai CV risk scores were categorized as follows: <10%

(low), 10% to <20% (moderate), 20% to <30% (high), and ≥30% (very high). High or very high CVD risk was defined as a 10-year predicted CVD risk of ≥ 20% [28].

Supplementary Table S2. Metrics for measurement and quantitative assessment of CVH [22]

CVH metric	Quantification of CVH metric	Level	Points
**Diet**	Mediterranean eating pattern (MEP)<br>MEP was assessed by 16 questions with scoring criteria of 0-16, with higher scores indicating higher reaching MEP[22].	MEP score 15-16<br>MEP score 12-14<br>MEP score 8-11<br>MEP score 4-7<br>MEP score 0-3	100<br>80<br>50<br>25<br>0
**Physical activity**	Minutes of moderate or greater intensity activity per week	≥150 minutes<br>120–149 minutes<br>90–119 minutes<br>60–89 minutes<br>30–59 minutes<br>1–29 minutes<br>0 minutes	100<br>90<br>80<br>60<br>40<br>20<br>0
**Nicotine exposure**	Combustible tobacco uses or inhaled nicotine delivery system (NDS) use; or secondhand smoke exposure	Never smoker<br>Former smoker, quit ≥5 y<br>Former smoker, quit 1–<5 y<br>Former smoker, quit <1 y, or currently using inhaled NDS<br>Current smoker	100<br>75<br>60<br>20<br><br>0
		Subtract 20 points (unless score is 0) for living with active indoor smoker in home	
**Sleep health**	Average hours of sleep per night	7–<9<br>9–<10<br>6–<7<br>5–<6 or ≥10<br>4–<5<br>0 <4	100<br>90<br>70<br>40<br>20<br>0
**Body mass index**	BMI (kg/m^2)	18.5–<23.0<br>23.0–<25.0<br>25.0–<30.0<br>30.0–<35.0<br>≥35.0	100<br>75<br>50<br>25<br>0
**Blood lipid**	Non–HDL cholesterol (mg/dL)	<130<br>130–159<br>160–189<br>190–219<br>≥220	100<br>60<br>40<br>20<br>0
		If drug-treated level, subtract 20 points	
**Blood glucose**	FBG (mg/dL) or HbA1c (%)	No history of diabetes and FBG <100 (or HbA1c <5.7)<br>No diabetes and FBG 100–125 (or HbA1c 5.7–6.4) (prediabetes)<br>Diabetes with HbA1c <7.0<br>Diabetes with HbA1c 7.0–7.9<br>Diabetes with HbA1c 8.0–8.9<br>Diabetes with Hb A1c 9.0–9.9<br>Diabetes with HbA1c ≥10.0	100<br>60<br>50<br>30<br>20<br>10<br>0
**Blood pressure**	SBP and DBP (mmHg)	<120/<80 (optimal)<br>120–129/<80 (elevated)<br>130–139 or 80–89 (stage 1 HTN)<br>140–159 or 90–99<br>0 ≥160 or ≥100	100<br>75<br>50<br>25<br>0
		Subtract 20 points if treated level	

Supplementary Table S3. Uses of antihypertensive medications for people with hypertension in rural Thailand

	Age-adjusted sex specific		Sex-adjusted age specific			Total
	Men	Women	20–44	45–64	≥65	
**Participants, N**	**317**	**683**	**49**	**443**	**508**	**1000**
**No medication use, weighted %**	**12.2**	**6.0**	**21.9**[†a,*b]	**7.5**	**7.0**	**8.3**
n	28	37	5	31	29	**65**
**Single therapy, weighted %**	**39.8**	**55.4**	**50.2**	**57.0**[*c]	**44.7**	**49.9**
n	144	366	31	234	245	510
ACEI/ARB	52.8	39.6	35.6	61.7	29.0	44.8
CCB	44.1	48.5	33.5	32.9	60.9	45.4
β-blocker	1.2	3.4	0	5.1	0.9	3.0
Diuretic	0.6	7.5	30.8	0.3	7.2	5.8
Other	1.3	0.9	0	0	2.0	0.9
**Dual therapy, weighted %**	**34.6**	**29.6**	**26.0**	**27.6**	**34.7**	**31.3**
n	119	232	11.0	151	189	351
ACEI/ARB + CCB	77.9	78.6	91.2	76.6	78.7	78.5
ACEI/ARB + β-blocker	1.3	2.7	1.3	3.2	1.5	2.1
ACEI/ARB + Diuretic	3.0	2.9	0	3.5	2.8	2.9
ACEI/ARB + Other	11.7	7.2	0	8.8	9.3	9.1
CCB + β-blocker	1.9	3.1	7.5	1.7	3.1	2.5
CCB + Diuretic	1.0	4.6	0	4.7	2.7	3.1
CCB + Other	1.7	0.6	0	1.3	0.8	1.0
β-blocker + Diuretic	0	0.3	0	0.3	0.2	0.2
β-blocker + Other	1.5	0	0	0	0.9	0.6
Diuretic + Other	0	0	0	0	0	0
**Polytherapy, weighted %**	**13.4**	**9.1**	**1.9**[*b]	**7.8**	**13.5**	**10.5**
n	26	48	2	27	45	74
ACEI/ARB + CCB +β-blocker	14.4	39.3	36.7	56.2	9.2	21.7
ACEI/ARB + CCB + Diuretic	70.7	34.4	0	23.8	69.6	58
ACEI/ARB + CCB + Other	8.8	4.5	0	4.3	7.9	7.2
CCB + β-blocker + Diuretic	1.2	3.6	0	1.4	3.6	2.3
CCB + β-blocker + Other	0	7.4	63.3	4.5	0.8	2.6
β-blocker + Diuretic+ Other	0	1.7	0	0.7	1.7	1.0
Other combinations	4.9	9.1	0	9.2	7.3	7.1
**Single pill combination**[‡]**, weighted %**	**1.0**	**0.1**[*]	**0**[†a, †b]	**0.3**	**0.4**	**0.5**
n	2	1	0	1	2	3
**Medication adherence scales**[§]						
n	**289**	**646**	**44**	**412**	**479**	**935**
Mean (SE)	33.2 (0.7)	34.3 (0.1)	33.2 (0.4)	34.2 (0.1)	33.8 (0.4)	33.9 (0.3)
Tertile 1 (12-34), %	59.5	46.1	75.0	48.3	49.7	51.2
Tertile 2 (35-35), %	30.2	30.7	17.1	30.3	32.1	30.2
Tertile 3 (36-36), %	10.3	23.1	7.8	21.3	18.2	18.6

SE: standard error of the mean

[*]$p<0.05$, [†]$p<0.001$

[‡]Individuals used dual therapy or polytherapy (n=425), [§]Individuals used antihypertensive medications (n=935)

[a]20–44 vs 45-64, [b]20–44 vs 65 and older, [c]45–64 vs 65 and older

No reported use of mineralocorticoid receptor antagonists.

Supplementary Table S4. Distribution of observed blood pressure of people with hypertension in rural Thailand, stratified by age group and sex

BP outcomes	Age-adjusted sex specific		Sex-adjusted age specific			Total
	**Men**	**Women**	**20-44**	**45-64**	**≥65**	
**Participants, N**	**317**	**683**	**49**	**443**	**508**	**1000**
**SBP (mmHg), weighted % (95% CI)**						
<120	10.6 (5.0–21.3)	21.5 (14.1–31.2)	9.7 (2.9–27.8)	22.3 (11.0–39.9)	15.1 (13.1–17.3)	17.5 (11.6–25.5)
120-129	38.8 (21.0–60.2)	21.2 (17.9–24.9)[*]	7.6 (2.1–23.9)[*b]	27.2 (14.7–44.7)	28.3 (24.4–32.5)	27.3 (20.3–35.5)
130-139	19.6 (13.8–27.1)	23.8 (21.0–27.0)	9.2 (2.8–26.1)	22.2 (15.6–30.6)	24.0 (17.3–32.4)	22.1 (18.2–26.7)
140-159	20.4 (13.9–28.8)	25.8 (18.2–35.1)	30.7 (8.9–66.7)	22.9 (20.0–26.1)	24.4 (14.7–37.8)	24.0 (21.5–26.7)
160-179	10 (6.6–14.9)	7.0 (5.7–8.5)	42.8 (9.3–84.5)[*a,*b]	5.2 (2.0–12.7)	7.1 (4.0–12.2)	8.5 (7.0–10.2)
≥180	0.6 (0.2–2.1)	0.7 (0.3–1.9)	n/a	0.3 (0.1–1.2)	1.1 (0.4–3.0)	0.7 (0.3–1.8)
weighted mean (SE)	134.6 (1.4)	134.3 (1.9)	145.5 (6.7)	131.6 (1.1)[‡c]	135.8 (1.2)	134.5 (1.0)
**DBP (mmHg), weighted % (95% CI)**						
<80	60.0 (42.5–75.3)	66.9 (58.1–74.6)	10.9 (3.1–31.8)[†a,‡b]	55.7 (45.7–65.2)[‡c]	77.8 (70.7–83.5)	63.6 (57.9–69.0)
80-84	22.0 (12.5–35.7)	11.7 (7.1–18.7)	9.0 (2.4–28.9)	18.9 (12.4–27.7)	12.1 (9.4–15.4)	15.4 (12.2–19.2)
85-89	4.7 (1.9–10.9)	10.2 (8.6–12.1)	5.4 (1.2–21.9)	13.5 (11.4–16)[†c]	4.3 (2.0–9.1)	8.2 (6.0–11.0)
90-99	8.8 (5.7–13.1)	8.8 (5.2–14.5)	53.9 (17.2–86.8)[**a,†b]	10.1 (6.8–14.8)[†c]	3.2 (1.3–7.5)	9.3 (5.3–15.9)
100-109	4.0 (1.1–13.4)	2.3 (0.9–5.8)	20.5 (4.2–60.5)[*a,‡b]	1.3 (0.5–3.7)	2.5 (0.7–8.1)	3.2 (1.1–9.0)
≥110	0.6 (0.1–5.2)	0.2 (0.0–0.7)	0.2 (0.0–2.3)	0.4 (0.1–1.8)	0.2 (0.0–1.7)	0.3 (0.1–1.6)
weighted mean (SE)	77.7 (2.0)	76.3 (0.7)	92.5 (2.3)[‡a,‡b]	78.7 (1.1)[‡c]	73.5 (0.7)	77.0 (0.6)
**Control BP, weighted % (95% CI)**						
<140/90 mmHg	67.1 (55.1–77.2)	63.0 (52.0–72.8)	20.8 (6.1–51.6)[*a,*b]	65.9 (61.3–70.2)	67.1 (56.0–76.6)	63.9 (59.4–68.1)
Optimal target	49.6 (35.3–63.9)	47.7 (33.7–62.1)	9.8 (2.8–28.8)[*a,†b]	37.9 (29.9–46.6)[‡c]	61.1 (51.9–69.6)	47.8 (39.8–55.9)
**HTN crisis, weighted % (95% CI)**						
≥180/110 mmHg	1.2 (0.3–4.6)	0.8 (0.3–2.0)	0.2 (0.0–2.3)	0.6 (0.2–1.9)	1.3 (0.5–3.4)	0.9 (0.4–2.4)

BP: blood pressure, SBP: systolic blood pressure, DBP: diastolic blood pressure, HTN: hypertension, SE: standard error of the mean, CI: confidence interval

Optimal target : SBP<130mmHg and DBP<80mmHg for age 20-64 years, SBP<140 mmHg and DBP<80 mmHg for aged 65 years and older

[*]p<0.05, [†]p<0.01, [‡]p<0.001

[a]20-44 vs 45-64, [b]20-44 vs 65 and older, [c]45-64 vs 65 and older

Supplementary Table S5. Distribution of observed blood pressure of people with hypertension in rural Thailand, stratified by geographical region

BP outcomes	Age- and sex-adjusted geographic region specific				Total
	Central	Northeast	North	South	
**Participants, N**	**254**	**246**	**252**	**248**	**1000**
**SBP (mmHg), weighted % (95% CI)**					
<120	14.6 (11.3–18.6)[†b]	20.9 (9.7–39.3)	25.1 (17.3–35.0)[†b]	11.4 (7.1–17.8)	17.5 (11.6–25.5)
120-129	26.6 (17.7–38.1)	27.8 (17.9–40.4)	28.5 (23.6–33.9)	20.5 (14.3–28.5)	27.3 (20.3–35.5)
130-139	22.4 (16.1–30.3)	19.2 (16.7–21.9)[*d,‡e]	25.5 (20.2–31.6)	29.5 (24.0–35.6)	22.1 (18.2–26.7)
140-159	28.7 (24.3–33.5)	23.5 (14.0–36.7)	17.3 (10.7–26.8)	27.2 (23.0–31.8)	24.0 (21.5–26.7)
160-179	6.0 (4.2–8.6)[*b,†c]	8.3 (4.2–15.6)[‡d]	2.6 (1.3–5.5)[†f]	11.0 (7.9–15.1)	8.5 (7.0–10.2)
≥180	1.6 (0.4–6.4)	0.4 (0.0–3.6)	1.0 (0.2–5.1)	0.5 (0.1–3.4)	0.7 (0.3–1.8)
weighted mean (SE)	135.8 (1.3)[†b]	133.7 (2.0)[*d]	129.8 (1.2)[‡f]	137.9 (0.8)	134.5 (1.0)
**DBP (mmHg), weighted % (95% CI)**					
<80	60.2 (50.3–69.4)[*a,*b]	70.9 (64.7–76.5)[‡e]	69.1 (65.5–72.5)[‡f]	51.1 (42.0–60.1)	63.6 (57.9–69.0)
80-84	15.6 (10.0–23.7)	11.4 (9.3–13.9)[†e]	14.8 (10.9–19.9)[*f]	21.4 (15.5–28.8)	15.4 (12.2–19.2)
85-89	12.4 (8.5–17.7)[*a]	6.8 (4.8–9.6)[†e]	8.0 (5.6–11.3)	11.1 (8.3–14.7)	8.2 (6.0–11.0)
90-99	8.5 (4.4–15.9)	7.2 (3.2–15.7)	7.9 (4.6–13.2)	12.8 (8.2–19.7)	9.3 (5.3–15.9)
100-109	2.7 (1.3–5.8)[†b]	3.6 (0.7–16.0)[‡d]	0[‡f]	2.3 (0.8–6.4)	3.2 (1.1–9.0)
≥110	0.6 (0.1–3.3)[†a]	0[‡e]	0.2 (0.0–2.4)	1.2 (0.1–9.9)	0.3 (0.1–1.6)
weighted mean (SE)	78.0 (0.7)[*a,†b]	75.6 (0.8)[*e]	75.3 (0.6)[†f]	79.4 (1.2)	77.0 (0.6)
**Control BP, weighted % (95% CI)**					
<140/90 mmHg	59.1 (49.5–68.0)[†b]	64.7 (54.2–74.0)[*d]	77.5 (67.0–85.4)[‡f]	57.5 (55.1–59.9)	63.9 (59.4–68.1)
Optimal target	41.5 (29.6–54.6)[†b]	53.1 (37.4–68.2)[†e]	57.6 (51.3–63.7)[‡f]	34.6 (30.6–38.7)	47.8 (39.8–55.9)
**HTN crisis, weighted % (95% CI)**					
≥180/110 mmHg	1.8 (0.5–5.9)	0.4 (0.0–3.6)	1.2 (0.3–4.7)	1.6 (0.3–7.6)	0.9 (0.4–2.4)

BP: blood pressure, SBP: systolic blood pressure, DBP: diastolic blood pressure, HTN: hypertension, SE: standard error of the mean, CI: confidence interval

Optimal target : SBP<130mmHg and DBP<80mmHg for age 20-64 years, SBP<140 mmHg and DBP<80 mmHg for aged 65 years and older

[*]p<0.05, [†]p<0.01, [‡]p<0.001

[a]Central vs Northeast, [b]Central vs North, [c]Central vs South, [d]Northeast vs North, [e]Northeast vs South, [f]North vs South

Supplementary Table S6. Univariable analysis for factors associated with hypertension control among people with hypertension in rural Thailand

Factors	Total N	HTN control (<140/90 mmHg)			HTN control (Optimal target)		
	n (%)	weighted %	unadjusted PR (95% CI)	*p*-value	weighted %	unadjusted PR (95% CI)	*p*-value
**Sex**							
Women	683 (68.3)	62.3	Ref.		47.1	Ref.	
Men	317 (31.7)	66.7	1.07 (0.81-1.42)	0.629	49.1	1.04 (0.61-1.78)	0.875
**Age, years**							
20-44	49 (4.9)	20.8	0.31 (0.1-0.93)	0.037	9.8	0.16 (0.05-0.55)	0.005
45-65	443 (44.3)	65.6	0.97 (0.8-1.17)	0.744	37.9	0.62 (0.55-0.69)	<0.001
≥65	508 (50.8)	67.6	Ref.		61.3	Ref.	
**Geographic region**							
Central	254 (25.4)	59.3	0.77 (0.65-0.90)	0.002	41.4	0.72 (0.55-0.94)	0.018
Northeast	246 (24.6)	63.7	0.82 (0.71-0.95)	0.011	52.1	0.91 (0.69-1.19)	0.471
North	252 (25.2)	77.4	Ref.		57.5	Ref.	
South	248 (24.8)	57.7	0.75 (0.67-0.83)	<0.001	34.6	0.60 (0.52-0.70)	<0.001
**Health insurance scheme**							
Universal health coverage	891 (89.1)	64.2	Ref.		48.2	Ref.	
Civil servant medical benefits	77 (7.7)	53.1	0.83 (0.63-1.09)	0.167	39.6	0.82 (0.62-1.08)	0.156
Social security	19 (1.9)	83.1	1.29 (0.98-1.70)	0.065	60.3	1.25 (0.63-2.49)	0.514
others	13 (1.3)	71	1.10 (0.75-1.63)	0.605	31.5	0.65 (0.43-0.99)	0.043
**Marital status**							
Married	674 (67.4)	66.6	Ref.		49.7	Ref.	
Widowed	225 (22.5)	66.5	1.00 (0.85-1.17)	0.981	54.6	1.10 (0.84-1.44)	0.486
Divorced/separated	51 (5.1)	46.5	0.70 (0.24-2.07)	0.506	17.8	0.36 (0.10-1.29)	0.113
Never married	50 (5.0)	45.4	0.68 (0.46-1.00)	0.050	36.7	0.74 (0.51-1.07)	0.102
**Educational attainment**							
Never attend	116 (11.6)	64.7	Ref.		52.2	Ref.	
Nonformal	4 (0.4)	89.2	1.38 (1.03-1.84)	0.030	71.8	1.38 (0.74-2.57)	0.308
Grade 1-6	725 (72.5)	68.4	1.06 (0.91-1.22)	0.440	52.4	1.00 (0.79-1.29)	0.969
Grade 7-9	60 (6.0)	35.0	0.54 (0.21-1.39)	0.196	16.4	0.31 (0.10-0.97)	0.045
Grade 10-12	58 (5.8)	41.7	0.64 (0.39-1.06)	0.083	25.6	0.49 (0.29-0.84)	0.010
Vocational	19 (1.9)	49.8	0.77 (0.5-1.17)	0.218	34.7	0.66 (0.37-1.21)	0.175
Bachelor's degree or higher	18 (1.8)	54.0	0.83 (0.5-1.39)	0.475	20.7	0.40 (0.16-0.98)	0.046
**Occupation**							
No occupation / Retired	462 (46.2)	68.4	Ref.		53.0	Ref.	
Homemaker	30 (3.0)	47.7	0.70 (0.40-1.22)	0.201	44.5	0.84 (0.53-1.34)	0.454
Mechanic	8 (0.8)	12.2	0.18 (0.03-1.16)	0.071	1.5	0.03 (0.00-0.33)	0.006
Seller	94 (9.4)	54.1	0.79 (0.69-0.91)	0.002	41.5	0.78 (0.52-1.19)	0.245
Service	15 (1.5)	58.7	0.86 (0.53-1.39)	0.523	21.8	0.41 (0.14-1.25)	0.113
Professional	5 (0.5)	75.0	1.10 (0.59-2.03)	0.762	58.6	1.11 (0.44-2.77)	0.826
Farming/Agriculture	351 (35.1)	70.6	1.03 (0.92-1.16)	0.572	48.5	0.92 (0.63-1.33)	0.639
Priest	4 (0.4)	71.9	1.05 (0.65-1.69)	0.832	6.8	0.13 (0.01-1.39)	0.089
Government officer	10 (1.0)	72.6	1.06 (0.70-1.62)	0.772	5.6	0.11 (0.02-0.60)	0.013
Others	21 (2.1)	45.0	0.66 (0.49-0.89)	0.007	39.0	0.74 (0.59-0.92)	0.010
**Household income, per year**							
Under 50,000 Baht	519 (51.9)	73.1	Ref.		57.2	Ref.	
50,000-99,999 Baht	221 (22.1)	52.1	0.71 (0.57-0.89)	0.004	35.1	0.61 (0.51-0.73)	<0.001
100,000-149,999 Baht	111 (11.1)	63.3	0.87 (0.73-1.03)	0.094	33.3	0.58 (0.44-0.77)	<0.001
150,000-199,999 Baht	34 (3.4)	32.8	0.45 (0.15-1.31)	0.139	21.8	0.38 (0.13-1.11)	0.075
200,000-249,999 Baht	40 (4.0)	50.4	0.69 (0.32-1.5)	0.340	43.3	0.76 (0.35-1.65)	0.472
250,000-299,999 Baht	6 (0.6)	51.4	0.70 (0.29-1.69)	0.420	51.4	0.90 (0.37-2.18)	0.808
300,000 Baht and over	44 (4.4)	43.8	0.60 (0.29-1.24)	0.163	24.5	0.43 (0.17-1.09)	0.074
No intention to respond	25 (2.5)	64.0	0.88 (0.62-1.23)	0.429	60.1	1.05 (0.83-1.33)	0.671
**Duration of hypertension treatment, years**							
1-3	202 (20.2)	53.1	Ref.		30.1	Ref.	
4-6	184 (18.4)	68.2	1.29 (0.90-1.84)	0.164	47.9	1.59 (0.81-3.12)	0.170
7-9	258 (25.8)	71.1	1.34 (0.89-2.01)	0.152	60.6	2.01 (0.94-4.29)	0.069
≥10	356 (35.6)	62.4	1.18 (0.91-1.52)	0.211	48.7	1.62 (0.91-2.87)	0.097
**Adding extra salt or a salty sauce before eating**							
1 time per week and lower	946 (94.6)	64.9	Ref.		49.0	Ref.	
2 -4 times per week and higher	54 (5.4)	40.3	0.62 (0.33-1.19)	0.144	21.1	0.43 (0.16-1.16)	0.093

Supplementary Table S6. Univariable analysis for factors associated with hypertension control among people with hypertension in rural Thailand (Continue)

Factors	Total N	HTN control (<140/90 mmHg)			HTN control (Optimal target)		
	n (%)	weighted %	unadjusted PR (95% CI)	*p*-value	weighted %	unadjusted PR (95% CI)	*p*-value
**Physical activity level**							
Low	36 (3.6)	19.1	0.29 (0.07-1.28)	0.100	16.3	0.34 (0.07-1.66)	0.177
Moderate	159 (15.9)	69.3	1.06 (0.89-1.26)	0.501	55.3	1.16 (0.76-1.77)	0.492
High	805 (80.5)	65.3	Ref.		47.8	Ref.	
**Sleep duration, hours**							
<8	352 (35.2)	57.2	Ref.		39.7	Ref.	
≥8	648 (64.8)	67.3	1.18 (0.73-1.91)	0.488	51.8	1.30 (0.63-2.68)	0.458
**Smoking status**							
Never smoker	704 (70.4)	61.9	Ref.		46.5	Ref.	
Former smoker	193 (19.3)	69.4	1.12 (0.77-1.62)	0.534	57.3	1.23 (0.71-2.14)	0.445
Someday smoker	24 (2.4)	83.8	1.35 (1.10-1.67)	0.006	29.4	0.63 (0.15-2.66)	0.521
Everyday smoker	79 (7.9)	63.2	1.02 (0.80-1.31)	0.863	43.0	0.93 (0.74-1.16)	0.499
**Alcohol use**							
Lifetime abstainer	676 (67.6)	66.9			52.0	Ref.	
Former drinker	140 (14.0)	64.7	0.97 (0.83-1.13)	0.680	51.8	1.00 (0.81-1.23)	0.969
Current infrequent/light drinker	112 (11.2)	56.2	0.84 (0.55-1.30)	0.423	31.3	0.60 (0.27-1.32)	0.198
Current moderate/heavier drinker	72 (7.2)	54.4	0.81 (0.53-1.24)	0.326	42.9	0.82 (0.44-1.53)	0.531
**No. of antihypertensive medication use**							
No medication use	65 (6.5)	54.2	0.89 (0.73-1.08)	0.232	34.5	0.85 (0.53-1.34)	0.463
Single therapy	510 (51.0)	61.1	Ref.		40.7	Ref.	
Dual therapy	351 (35.1)	67.2	1.10 (0.83-1.45)	0.489	55.5	1.36 (0.97-1.92)	0.077
Poly therapy	74 (7.4)	74.8	1.22 (0.99-1.52)	0.065	68.9	1.69 (1.31-2.19)	<0.001
**Type 2 diabetes**							
No	694 (69.4)	67.8			52.0	Ref.	
Yes	306 (30.6)	56.0	0.83 (0.56-1.22)	0.326	39.3	0.76 (0.44-1.3)	0.303
**Hyperlipidemia**							
No	36 (3.6)	76.7			29.5	Ref.	
Yes	964 (96.4)	63.4	0.83 (0.58-1.17)	0.274	48.5	1.64 (0.60-4.49)	0.323
**Chronic kidney disease**							
No	864 (86.4)	63.5			46.8	Ref.	
Yes	136 (13.6)	66.1	1.04 (0.73-1.47)	0.816	53.7	1.15 (0.64-2.07)	0.634
**Body mass index, kg/m^2**							
18.5−<23.0	284 (28.4)	73.0	Ref.		57.9	Ref.	
<18.5	59 (5.9)	75.9	1.04 (0.87-1.25)	0.664	67.0	1.16 (0.94-1.43)	0.172
23.0−<25.0	176 (17.6)	73.1	1.00 (0.87-1.15)	0.987	48.1	0.83 (0.73-0.95)	0.007
25.0−<30.0	321 (32.1)	57.2	0.78 (0.60-1.02)	0.072	38.3	0.66 (0.56-0.79)	<0.001
≥30	160 (16.0)	52.0	0.71 (0.57-0.90)	0.005	42.5	0.73 (0.67-0.81)	<0.001
**Waist circumference, cm**							
<90 in men and <80 in women	368 (36.8)	72.2	Ref.		57.7	Ref.	
≥90 in men and ≥80 in women	632 (63.2)	58.2	0.81 (0.68-0.95)	0.013	41.1	0.71 (0.59-0.85)	0.001
**Waist to hip ratio**							
<0.90 in men and <0.85 in women	260 (26.0)	72.5	Ref.		58.9	Ref.	
≥0.90 in men and ≥0.85 in women	740 (74.0)	60.1	0.83 (0.66-1.05)	0.112	43.0	0.73 (0.5-1.07)	0.105
**Psycological stress**							
Low	609 (60.9)	61.4	Ref.		44.6	Ref.	
Moderate	383 (38.3)	66.9	1.09 (0.90-1.32)	0.362	51.5	1.15 (0.91-1.46)	0.229
High	8 (0.8)	53.0	0.86 (0.36-2.09)	0.740	43.9	0.98 (0.35-2.73)	0.973
**Depression**							
non-minimal	852 (85.2)	60.9	Ref.		44.3	Ref.	
Milde	136 (13.6)	78.9	1.30 (0.97-1.64)	0.077	65.3	1.47 (1.22-1.78)	<0.001
Moderate to moderately severe	12 (1.2)	60.9	1.00 (0.47-1.54)	0.995	45.5	1.02 (0.35-1.69)	0.956
**General anxiety**							
Minimal	940 (94.0)	61.5			45.9	Ref.	
Mild	55 (5.5)	89.9	1.46 (1.19-1.79)	0.001	67.8	1.48 (1.26-1.72)	<0.001
Moderate to severe	5 (0.5)	94.6	1.54 (1.31-1.77)	<0.001	94.8	2.05 (1.64-2.46)	<0.001

SBP: systolic blood pressure, DBP: diastolic blood pressure, HTN: hypertension, PR: prevalence ratio, CI: confidence interval

Optimal target : SBP<130mmHg and DBP<80mmHg for age 20-64 years, SBP<140 mmHg and DBP<80 mmHg for aged 65 years and older

Supplementary Table S7. Cardiovascular health score among people with hypertension in rural Thailand, stratified by sex and age group

CVH metrics	Age-adjusted sex specific		Sex adjusted age specific			Total
	**Men**	**Women**	**20-44**	**45-64**	**≥65**	
**Participants, N**	**317**	**683**	**49**	**443**	**508**	**1000**
**Diet**						
weighted mean (SE)	33.0 (1.4)	30.5 (0.8)	30.6 (3.5)	31.2 (0.7)	31.5 (1.3)	31.4 (0.7)
**Physical activity**						
weighted mean (SE)	91.7 (4.6)	95.3 (1.3)	99.8 (0.2)[*b]	97.0 (1.7)[†c]	91.1 (3.1)	94.1 (2.6)
**Nicotin exposure**						
weighted mean (SE)	52.2 (2.7)	90.6 (1.0)[‡]	87.1 (7.3)	75.0 (1.8)	80.5 (3.1)	76.8 (0.9)
**Sleep health**						
weighted mean (SE)	88.7 (2.0)	84.2 (1.3)[†]	90.0 (3.7)	86.0 (2.2)	84.8 (1.1)	85.9 (1.5)
**Body mass index**						
weighted mean (SE)	70.5 (5.0)	60.1 (1.5)[*]	31.4 (4.7)[‡a,‡b]	54.8 (2.0)[‡c]	74.0 (3.6)	63.4 (1.9)
**Blood lipids**						
weighted mean (SE)	67.2 (3.6)	61.6 (2.8)[†]	49.9 (15.0)	64.1 (3.4)	64.1 (2.0)	63.5 (3.2)
**Blood glucose**						
weighted mean (SE)	77.1 (4.2)	71.6 (6.4)[*]	48.6 (21.0)	71.2 (5.5)[*c]	77.6 (4.6)	73.3 (6.1)
**Blood pressure**						
weighted mean (SE)	36.1 (2.8)	35.8 (3.3)	12.0 (6.4)[†a,†b]	37.4 (2.3)	36.9 (2.2)	35.6 (1.8)
**Total CVH Score**						
weighted mean (SE)	64.6 (0.7)	66.2 (1.4)	56.2 (4.6)[*a,*b]	64.6 (1.3)[*c]	67.6 (0.3)	65.5 (1.0)
**CVH level, weighted % (95% CI)**						
Poor	12.9 (9.0–18.0)	5.7 (4.5–7.1)[†]	50.6 (14.6–86.0)[*a,*b]	7.5 (5.8–9.5)	4.3 (2.0–8.7)	8.8 (5.9–13.0)
Moderate	77.5 (70.1–83.5)	87.4 (84.8–89.7)[‡]	45.8 (13.0–82.8)[*a,*b]	84.7 (80.0–88.5)[*c]	87.6 (84.3–90.3)	83.3 (77.8–87.7)
High	9.7 (7.0–13.1)	6.9 (4.9–9.6)[*]	3.6 (0.7–15.3)	7.8 (5.3–11.4)	8.1 (5.7–11.4)	7.8 (5.9–10.4)

CVH: cardiovascular health, SE: standard error, CI: confidence interval

[*]p<0.05, [†]p<0.01, [‡]p<0.001

[a]20-44 vs 45-64, [b]20-44 vs 65 and older, [c]45-64 vs 65 and older

Supplementary Table S8. Cardiovascular health score among people with hypertension in rural Thailand, stratified by geographical region

CVH metrics	Age- and sex-adjusted geographic region specific				Total
	Central	Northeast	North	South	
**Participants, N**	**254**	**246**	**252**	**248**	**1000**
**Diet**					
weighted mean (SE)	32.2 (2.6)	30.8 (1.1)	30.4 (0.7)†f	31.8 (0.5)	31.4 (0.7)
**Physical activity**					
weighted mean (SE)	93.5 (2.4)	92.8 (4.2)	97.7 (0.9)	95.8 (0.9)	94.1 (2.6)
**Nicotin exposure**					
weighted mean (SE)	78.1 (2.0)	79.5 (2.1)	77.1 (1.8)	77.6 (1.5)	76.8 (0.9)
**Sleep health**					
weighted mean (SE)	86.1 (2.2)†c	87.3 (2.3)†e	90.4 (1.5)‡f	77.6 (2.3)	85.9 (1.5)
**Body mass index**					
weighted mean (SE)	58.2 (1.9)‡b	62.0 (4.0)†d	76.3 (2.3)‡f	61.4 (2.9)	63.4 (1.9)
**Blood lipids**					
weighted mean (SE)	66.2 (2.8)	60.6 (5.7)	65.7 (0.9)	66.7 (2.0)	63.5 (3.2)
**Blood glucose**					
weighted mean (SE)	63.6 (3.2)‡c	71.3 (11.0)	73.6 (5.6)*f	85.0 (2.2)	73.3 (6.1)
**Blood pressure**					
weighted mean (SE)	32.1 (2.2)*a,‡b	38.6 (3.2)‡e	42.6 (2.4)‡f	27.8 (0.9)	35.6 (1.8)
**Total CVH Score**					
weighted mean (SE)	63.8 (0.9)‡b,*c	65.4 (1.6)†d	69.2 (0.4)‡f	65.4 (0.4)	65.5 (1.0)
**CVH level, weighted % (95% CI)**					
Poor	7.1 (3.6–13.4)*b	10.0 (8.8–11.3)†d,†e	2.1 (0.7–6.1)	5.7 (3.8–8.4)	8.8 (5.9–13.0)
Moderate	88.5 (80.2–93.6)*a	81.0 (79.2–82.8)†e	85.2 (79.0–89.8)	90.9 (84.4–94.8)	83.3 (77.8–87.7)
High	4.4 (2.0–9.4)*a,*b	9.0 (7.5–10.7)*e	12.8 (7.0–22.2)*f	3.4 (1.0–10.7)	7.8 (5.9–10.4)

CVH: cardiovascular health, SE: standard error, CI: confidence interval

*p<0.05, †p<0.01, ‡p<0.001

[a]Central vs Northeast, [b]Central vs North, [c]Central vs South, [d]Northeast vs North, [e]Northeast vs South, [f]North vs South

Supplementary Table S9. Prevalence of cardiovascular diseases among people with hypertension in rural Thailand, stratified by sex and age group

	**Age-adjusted sex specific**		**Sex adjusted age specific**			**Total**
	**Men**	**Women**	**20-44**	**45-64**	**≥65**	
**Participants, N**	**317**	**683**	**49**	**443**	**508**	**1000**
**Stroke, weighted % (95% CI)**						
Overall	12.7 (6.1–24.6)	9.1 (4–19.2)	1.4 (0.1–13.4)	14.9 (6.6–30.2)	7.1 (4.1–11.9)	10.3 (7.4–14.2)
Self-reported history	11.8 (5.3–24.1)	7.0 (3.7–13.1)	1.4 (0.1–13.4)	11.9 (5.8–22.9)	6.3 (3.5–11.0)	8.7 (5.8–12.9)
ICD-10 (I60–I64)	5.9 (2.7–12.6)	2.6 (0.9–7.8)	0[‡a,‡b]	5.9 (3.0–11.0)	2.1 (0.8–5.2)	3.8 (2.7–5.3)
**Ischemic stroke, weighted % (95% CI)**						
Overall	12.2 (5.7–24.3)	5.4 (3.5–8.2)	1.4 (0.1–13.4)	9.6 (4.8–18.3)	6.4 (3.6–11)	7.8 (4.6–12.9)
Self-reported history	11.8 (5.3–24.1)	5.3 (3.4–8.2)	1.4 (0.1–13.4)	9.4 (4.6–18.3)	6.1 (3.4–10.7)	7.5 (4.3–12.8)
ICD-10 (I63)	1.0 (0.2–3.9)	0.3 (0.1–1.4)	0[‡a,‡b]	0.2 (0.0–1.4)	0.9 (0.3–2.8)	0.5 (0.2–1.7)
**Hemorrhagic stroke, weighted % (95% CI)**						
Overall	0.1 (0.0–0.9)	2.1 (0.5–8.1)[*]	0[‡a,‡b]	2.8 (0.5–13.5)	0.4 (0.1–1.7)	1.4 (0.4–4.7)
Self-reported history	0.0 (0.0–0.1)	1.8 (0.3–8.9)[†]	0[‡a,‡b]	2.5 (0.4–14.5)	0.2 (0.0–1.7)	1.1 (0.2–5.2)
ICD-10 (I60–I62)	0.1 (0.0–0.9)	0.3 (0.1–1.4)	0[‡a,‡b]	0.3 (0.0–2.3)	0.2 (0.0–1.0)	0.2 (0.1–0.9)
**Stroke unspecified, weighted % (95% CI)**						
ICD-10 (I64)	4.8 (1.8–12.2)	2.1 (0.5–8.4)	0[‡a,‡b]	5.6 (2.7–11.1)[*c]	1.0 (0.4–2.7)	3.1 (1.9–5.0)
**Ischemic heart disease, weighted % (95% CI)**						
Overall	2.9 (1.0–7.9)	0.6 (0.2–1.7)[†]	1.0 (0.1–10.0)	0.6 (0.2–2.0)	2.1 (0.8–5.2)	1.4 (0.6–3.5)
Self-reported history	2.0 (0.6–6.0)	0.4 (0.1–1.1)[‡]	1.0(0.1–10.0)	0.4 (0.1–1.8)	1.3 (0.4–3.7)	0.9 (0.4–2.5)
ICD-10	1.8 (0.5–6.4)	0.2 (0.0–0.9)[†]	1.0 (0.1–10.0)	0.4 (0.1–1.8)	1.0 (0.3–3.4)	0.8 (0.2–2.5)
ECG finding	0.8 (0.2–3.3)	0.3 (0.0–1.4)	1.0 (0.1–10.0)	0.2 (0.0–1.0)	0.6 (0.1–3.3)	0.4 (0.1–1.8)
**Atrial fribrillation, weighted % (95% CI)**						
Overall	3.0 (0.6–14.7)	0.1 (0.0–0.9)[*]	0[‡a,‡b]	2.0 (0.3–11.3)	0.3 (0.1–1.4)	1.2 (0.3–5.1)
Self-reported history	0.2 (0.0–1.8)	0.1 (0.0–1.0)	0	0	0.3 (0.1–1.4)	0.2 (0.0–0.7)
ECG finding	2.8 (0.5–15.5)	0.0 (0.0–0.1)[†]	0[‡a]	2.0 (0.3–11.3)[‡c]	0.0 (0.0–0.2)	1.0 (0.2–5.6)
**ECG-LVH, weighted % (95% CI)**						
Overall	6.4 (2.8–14)	5.9 (3.6–9.3)	2.2 (0.3–14.3)	4.3 (1.9–9.4)[*b]	7.9 (4.9–12.3)	6.0 (3.4–10.4)
Peguero-Lo Presti criteria	3.9 (1.6–9.4)	4.4 (2.5–7.6)	1.8 (0.2–15.4)	2.7 (1.2–6.1)	5.8 (3.4–9.8)	4.2 (2.3–7.4)
Cornell voltage index	2.2 (0.8–6.0)	2.4 (1.1–5.3)	0[‡a,‡b]	2.7 (1.1–6.8)	2.3 (0.9–5.4)	2.3 (1.0–5.1)
Sokolow-Lyon criteria	2.5 (1.0–6.1)	0.5 (0.1–1.9)[*]	0.4 (0.0–4.0)	0.3 (0.1–1.6)[*b]	1.9 (0.8–4.6)	1.2 (0.5–2.7)

ECG: electrocardiography, ECG-LVH: electrocardiographic-left ventricular hypertrophy, ICD-10: the 10th revision of the International Classification of Diseases, CI: confidence interval

[*]p<0.05, [†]p<0.01, [‡]p<0.001

[a]20-44 vs 45-64, [b]20-44 vs 65 and older, [c]45-64 vs 65 and older

Supplementary Table S10. Prevalence of cardiovascular diseases among people with hypertension in rural Thailand, stratified by geographical region

	**Age- and sex-adjusted geographic region specific**				**Total**
	**Central**	**Northeast**	**North**	**South**	
**Participants, N**	**254**	**246**	**252**	**248**	**1000**
**Stroke, weighted % (95% CI)**					
Overall	8.7 (5.2–14.4)	14.4 (9.4–21.5)[‡d,†e]	3.7 (2.4–5.8)	7.3 (4.6–11.4)	10.3 (7.4–14.2)
Self-reported history	6.4 (3.4–11.7)[*a]	12.0 (10.3–14.1)[‡d,†e]	3.7 (2.4–5.8)	5.8 (3.6–9.3)	8.7 (5.8–12.9)
ICD-10 (I60–I64)	5.0 (2.1–11.5)	4.3 (2.4–7.8)	1.5 (0.3–6.1)	2.7 (0.7–9.0)	3.8 (2.7–5.3)
**Ischemic stroke, weighted % (95% CI)**					
Overall	7.4 (3.8–13.8)	9.7 (6.1–14.9)[*d]	3.7 (2.4–5.8)	5.8 (3.4–9.7)	7.8 (4.6–12.9)
Self-reported history	6.4 (3.4–11.7)	9.7 (6.1–14.9)[*d]	3.7 (2.4–5.8)	5.3 (3.2–8.8)	7.5 (4.3–12.8)
ICD-10 (I63)	1.0 (0.1–8.7)[‡a]	0[‡d,‡e]	0.7 (0.1–3.5)	1.5 (0.3–7.0)	0.5 (0.2–1.7)
**Hemorrhagic stroke, weighted % (95% CI)**					
Overall	1.5 (0.5–4.4)	2.4 (0.3–16.7)	0.2 (0–2.8)	0.5 (0.0–4.6)	1.4 (0.4–4.7)
Self-reported history	0.1 (0.0–1.4)[*a,‡b]	2.4 (0.3–16.7)[‡d]	0[‡f]	0.5 (0.0–4.6)	1.1 (0.2–5.2)
ICD-10 (I60–I62)	1.4 (0.4–4.5)[‡a,‡c]	0[‡d]	0.2 (0.0–2.8)[‡f]	0	0.2 (0.1–0.9)
**Stroke unspecified, weighted % (95% CI)**					
ICD-10 (I64)	3.1 (0.9–10.1)	4.3 (2.4–7.8)[*d,*e]	0.6 (0.1–3.8)	1.2 (0.3–4.3)	3.1 (1.9–5.0)
**Ischemic heart disease, weighted % (95% CI)**					
Overall	6.5 (5.0–8.6)[‡a,‡b]	0.2 (0.0–1.2)[‡d,*e]	0[*f]	2.7 (0.7–9.1)	1.4 (0.6–3.5)
Self-reported history	3.5 (1.2–9.9)[†a,‡b]	0.2 (0.0–1.2)[‡d,*e]	0[‡f]	2.1 (0.5–8.0)	0.9 (0.4–2.5)
ICD-10	2.7 (0.7–10)[‡a,‡b]	0[‡e]	0[‡f]	2.2 (0.4–10.3)	0.8 (0.2–2.5)
ECG finding	3.9 (1.7–8.6)[‡a,‡b,‡c]	0	0	0	0.4 (0.1–1.8)
**Atrial fribrillation, weighted % (95% CI)**					
Overall	0.5 (0.1–4.6)[‡b]	1.6 (0.2–10.5)[‡d]	0[‡f]	0.4 (0.0–3.8)	1.2 (0.3–5.1)
Self-reported history	0.5 (0.1–4.6)[‡a,‡b]	0[‡e]	0[‡f]	0.4 (0.0–3.8)	0.2 (0.0–0.7)
ECG finding	0[‡a]	1.6 (0.2–10.5)[‡d,‡e]	0	0	1.0 (0.2–5.6)
**ECG-LVH, weighted % (95% CI)**					
Overall	4.5 (2.7–7.5)[†b,‡c]	2.9 (1.0–8.2)[*d,*e]	10.6 (7.5–14.9)	12.1 (9.9–14.7)	6.0 (3.4–10.4)
Peguero-Lo Presti criteria	1.2 (0.3–4.2)[†b,†c]	2.6 (0.9–7.9)	8.4 (6.0–11.7)	7.9 (4.7–12.8)	4.2 (2.3–7.4)
Cornell voltage index	1.8 (0.5–7.0)[*c]	0.3 (0.0–2.2)[*d,†e]	3.3 (1.5–7.2)[*f]	7.1 (4.5–11.1)	2.3 (1.0–5.1)
Sokolow-Lyon criteria	2.5 (1.0–6.0)[†a]	0.2 (0.0–1.2)[†d,†e]	2.1 (0.9–5.1)	2.3 (1.0–5.6)	1.2 (0.5–2.7)

ECG: electrocardiography, ECG-LVH: electrocardiographic-left ventricular hypertrophy, ICD-10: the 10th revision of the International Classification of Diseases, CI: confidence interval

[*]p<0.05, [†]p<0.01, [‡]p<0.001

[a]Central vs Northeast, [b]Central vs North, [c]Central vs South, [d]Northeast vs North, [e]Northeast vs South, [f]North vs South

Supplementary Table S11. Characteristics of people with hypertension aged 40-74 without a history of cardiovascular disease (ischemic heart disease and stroke) in rural Thailand

**Characteristics**	**Geographical region**				**Total**
	**Central**	**Northeast**	**North**	**South**	
**Participants, N**	**183**	**179**	**197**	**179**	**738**
**Sex, n (%)**					
Men	52 (28.4)	54 (30.2)	67 (34.0)	46 (25.7)	219 (29.7)
Women	131 (71.6)	125 (69.8)	130 (66)	133 (74.3)	519 (70.3)
**Age (years), n (%)**					
40-49	19 (10.4)	15 (8.4)	17 (8.6)	20 (11.2)	71 (9.6)
50-59	43 (23.5)	56 (31.3)	53 (26.9)	59 (33.0)	211 (28.6)
60-74	121 (66.1)	108 (60.3)	127 (64.5)	100 (55.9)	456 (61.8)
mean (SD)	61.5 (8.1)	61.7 (8.3)	61.7 (8.2)	60.6 (8.4)	61.4 (8.2)
**Current smoker, n (%)**					
No	163 (89.1)	163 (91.1)	171 (86.8)	158 (88.3)	655 (88.8)
Yes	20 (10.9)	16 (8.9)	26 (13.2)	21 (11.7)	83 (11.3)
**Diabetes, n (%)**					
No	90 (49.2)	101 (56.4)	164 (83.3)	147 (82.1)	502 (68.0)
Yes	93 (50.8)	78 (43.6)	33 (16.8)	32 (17.9)	236 (32.0)
**Systolic blood pressure, mmHg**					
mean (SD)	136.7 (16.5)	134.7 (16.4)	130.3 (15.8)	136.1 (14.8)	134.4 (16)
**Body mass index (kg/m^2)**					
mean (SD)	27.3 (4.9)	25.3 (4.5)	24.0 (3.7)	26.5 (5.2)	25.8 (4.8)
**Total cholesterol (mmol/L)**					
mean (SD)	4.7 (0.9)	4.7 (1.0)	4.7 (0.8)	4.7 (0.9)	4.7 (0.9)

SD: standard deviation

Supplementary Table S12. Predicted 10-year cardiovascular disease risk by the World Health Organization cardiovascular disease risk chart for people with hypertension aged 40-74 without a history of cardiovascular disease in rural Thailand

	Weighted mean (SE), %	Weighted % (95% CI)				
		Very low	Low	Moderate	High	Very high
		<5%	5%−< 10%	10%−< 20%	20%−< 30%	≥ 30%
**Laboratory-based**						
**Total**	9.9 (0.4)	19.8 (16.3–23.9)	34.1 (30.2–38.2)	38.2 (33.6–43.1)	7.6 (4.5–12.6)	0.3 (0.1–1.5)
**Sex***						
Men	11.7 (0.3)	13.2 (9.1–18.7)	26.2 (20.4–33.0)	47.9 (42.9–53.0)	11.7 (7.7–17.3)	1.0 (0.2–4.5)
Women	9.0 (0.4)	23.7 (21.0–26.6)	36.5 (30.8–42.6)	34.8 (29.7–40.2)	5.0 (1.9–12.4)	0
**Age (years)†**						
40–49	4.7 (0.9)	53.1 (23.2–80.9)	44.2 (17.9–74.2)	2.7 (0.4–17.4)	0	0
50–59	6.0 (0.5)	53.9 (39.3–67.9)	33.6 (16.2–56.9)	8.4 (4.9–14.1)	4.1 (1.1–13.6)	0
60–74	12.3 (0.3)	0.1 (0.0–0.5)	31.7 (25.1–39.2)	58.3 (52.0–64.3)	9.4 (6.5–13.4)	0.5 (0.1–2.2)
**Geographical region‡**						
Central	10.6 (0.3)	13.6 (9.3–19.5)	32.6 (25.7–40.4)	47.5 (40.2–54.9)	5.1 (2.0–12.6)	1.2 (0.2–6.4)
Northeast	10.3 (0.4)	20.4 (15.9–25.7)	29.0 (24.3–34.0)	40.8 (35.1–46.8)	9.8 (6.0–15.8)	0
North	8.6 (0.4)	26.8 (21.0–33.5)	37.9 (34.4–41.5)	31.0 (26.9–35.4)	3.6 (1.4–8.8)	0.8 (0.1–7.6)
South	8.6 (0.3)	21.3 (18.0–25.1)	41.0 (32.3–50.3)	34.6 (28.4–41.4)	3.1 (0.8–11.5)	0
**Non-laboratory-based**						
**Total**	9.1 (0.4)	18.7 (15.5–22.4)	40.7 (36.1–45.4)	37.5 (32.3–43)	3 (1.1–7.7)	0.2 (0.0–1.4)
**Sex***						
Men	11.5 (0.5)	12.1 (8.1–17.6)	27.6 (17.3–41.0)	51.5 (37.6–65.3)	8.2 (3.1–20.0)	0.5 (0.1–4.3)
Women	8.0 (0.1)	20.2 (15.1–26.5)	47.5 (38.4–56.9)	31.8 (27.0–37.0)	0.4 (0.1–1.7)	0
**Age (years)†**						
40–49	3.6 (0.4)	86.7 (73.3–93.9)	10.6 (5.1–20.5)	2.7 (0.4–17.4)	0	0
50–59	5.4 (0.2)	33.1 (22.9–45.2)	61.2 (53.0–68.8)	5.7 (2.6–12.1)	0	0
60–74	11.5 (0.4)	0	37.4 (29.9–45.6)	57.9 (50.8–64.7)	4.4 (1.8–10.4)	0.3 (0.0–2.1)
**Geographical region‡**						
Central	9.2 (0.3)	16.2 (11.0–23.1)	45.4 (39.0–52.0)	34.4 (29.9–39.3)	2.8 (1.0–7.5)	1.2 (0.2–6.4)
Northeast	9.2 (0.4)	18.2 (10.5–29.7)	36.0 (23.0–51.5)	42.9 (40.7–45.2)	2.9 (0.6–13.1)	0
North	8.3 (0.3)	20.7 (16.3–25.9)	47.6 (42.3–53.0)	28.8 (25.5–32.4)	2.9 (0.4–19.0)	0
South	8.9 (0.2)	17.2 (13.9–21.2)	46.6 (41.7–51.5)	34.2 (27.9–41.2)	2.0 (0.2–14.2)	0

*age-adjusted, †Sex-adjusted, ‡age- and sex-adjusted

Supplementary Table S13. Pearson's correlation coefficient of the predicted 10-year CVD risk using the non-laboratory-based and laboratory-based 2019 World Health Organization CVD risk score

	Correlation coefficient (95% CI)	*p*-value
**Overall** (n= 738)	0.88 (0.86–0.89)	<0.0001
**Sex**		
Men (n =219)	0.89 (0.86–0.92)	<0.001
Women (n =519)	0.86 (0.84–0.88)	<0.001
**Age (years)**		
40–49 (n = 71)	0.68 (0.53–0.79)	<0.001
50–59 (n = 211)	0.73 (0.66–0.79)	<0.001
60–74 (n = 456)	0.81 (0.77–0.84)	<0.001

Supplementary Table S14. Prevalence of high or very high predicted 10-year cardiovascular disease risk among people with hypertension aged 40−74 without a history of cardiovascular disease in rural Thailand (2019 World Health Organization CVD risk score)

	Weighted prevalence (95% CI)	
	**Laboratory-based**	**Non-Laboratory based**
**Total**	7.9 (4.9–12.5)	3.2 (1.2–7.8)
**Sex**[k]		
Men	12.7 (9.0–17.5)‡a	8.8 (3.5–20.1)‡a
Women	5.0 (1.9–12.4)	0.4 (0.1–1.7)
**Age (years)**[l]		
40–49	0‡b, ‡c	0‡c
50–59	4.1 (1.1–13.6)	0‡d
60–74	9.9 (7.3–13.4)	4.7 (2.0–10.5)
**Geographical region**[m]		
Central	6.3 (3.5–10.8)	4.0 (2.8–5.7)
Northeast	9.8 (6.0–15.8)†h,*i	2.9 (0.6–13.1)
North	4.3 (2.3–7.9)	2.9 (0.4–19.0)
South	3.1 (0.8–11.5)	2.0 (0.2–14.2)

*p<0.05, †p<0.01, ‡p<0.001

[a]Men vs Women

[b]40-49 vs 50-59, [c]40-49 vs 60-74, [d]50-59 vs 60-74

[e]Central vs Northeast, [f]Central vs North, [g]Central vs South, [h]Northeast vs North, [i]Northeast vs South, [j]North vs South

[k]age-adjusted, [l]sex-adjusted, [m]age- and sex-adjusted

Supplementary Table S15. Predicted 10-year cardiovascular disease risk by the Thai cardiovascular risk score for people with hypertension aged 30−70 without a history of cardiovascular disease in rural Thailand (N= 643)

	Weighted mean (SE), %	Weighted % (95% CI)			
		Low	Moderate	High	Very high
		**< 10%**	**10%−< 20%**	**20%−< 30%**	**≥ 30%**
**Laboratory-based**					
**Total**	15.6 (0.4)	38.5 (35.1–42.0)	28.1 (24.7–31.8)	18.6 (15.5–22.1)	14.9 (11.8–18.5)
**Sex***					
Men	20.3 (0.6)	19.9 (16.8–23.3)	29.2 (15.9–47.3)	22.7 (17.2–29.3)	28.2 (18.4–40.6)
Women	15.1 (0.6)	36.5 (33.6–39.6)	33.1 (26.5–40.3)	20.3 (15.6–26)	10.1 (5.9–16.8)
**Age (years)†**					
30–49	6.9 (1.0)	94.5 (83.0–98.4)	1.2 (0.3–5.5)	2.7 (0.4–17.4)	1.5 (0.2–12.3)
50–59	10.3 (0.7)	67.5 (58.8–75.2)	19.5 (9.8–35.2)	8.0 (4.4–13.9)	5.0 (1.8–13)
60–70	10.3 (0.7)	5.2 (2.6–9.9)	42.4 (33.7–51.7)	29.9 (22.9–38)	22.5 (18.7–26.9)
**Geographical region‡**					
Central	19.1 (0.6)	23.1 (17.4–29.9)	22.7 (16.5–30.4)	35.4 (23.4–49.5)	18.9 (13.7–25.5)
Northeast	17.2 (0.3)	31.6 (29.5–33.7)	28.8 (22.7–35.7)	21.5 (15.0–29.7)	18.2 (16.4–20.1)
North	14.6 (0.8)	38.9 (35.7–42.1)	33.7 (25.7–42.7)	16.7 (12.8–21.6)	10.7 (4.4–23.8)
South	14.6 (0.8)	32.6 (27.0–38.8)	41.2 (36.9–45.6)	15.4 (9.9–23.1)	10.8 (6.9–16.5)
**Non-laboratory-based**					
**Total**	20.1 (0.5)	18.2 (12.9–25.0)	30.9 (24.4–38.3)	20.4 (16.5–25.1)	30.5 (24.4–37.4)
**Sex***					
Men	22.0 (0.8)	13.5 (9.2–19.3)	24.7 (21.7–28.1)	20.7 (9.1–40.3)	41.1 (24.4–60.1)
Women	21.5 (0.8)	13.0 (6.3–24.8)	29.8 (21.7–39.5)	24.4 (20.7–28.6)	32.7 (27.2–38.8)
**Age (years)†**					
30–49	10.4 (1.4)	53.2 (23.6–80.7)	41.6 (14.9–74.3)	3.7 (0.8–15.6)	1.5 (0.2–12.3)
50–59	14.9 (0.9)	26.1 (16.8–38.2)	53.0 (46.6–59.3)	12.6 (8.0–19.2)	8.3 (4.6–14.8)
60–70	14.9 (0.9)	0.9 (0.3–2.6)	14.9 (9.5–22.6)	31.3 (26.3–36.7)	52.9 (43.4–62.3)
**Geographical region‡**					
Central	23.8 (0.6)	10.8 (6.6–17.2)	16.0 (13.0–19.6)	25.3 (20.5–30.8)	47.9 (38.7–57.1)
Northeast	22.7 (0.3)	8.5 (2.6–24.5)	32.0 (21.0–45.5)	22.4 (18.2–27.3)	37.0 (30.0–44.6)
North	18.4 (0.6)	24.8 (17.9–33.4)	29.8 (22.9–37.7)	20.3 (11.1–34.3)	25.1 (14.8–39.2)
South	21.0 (0.8)	38.9 (35.7–42.1)	33.7 (25.7–42.7)	16.7 (12.8–21.6)	10.7 (4.4–23.8)

*age-adjusted, †Sex-adjusted, ‡age- and sex-adjusted

Supplementary Table S16. Pearson's correlation coefficient of the predicted 10-year CVD risk using the
non-laboratory-based and laboratory-based Thai cardiovascular risk score

	Correlation coefficient (95% CI)	*p*-value
**Overall** (n = 643)	0.89 (0.87–0.90)	<0.0001
**Sex**		
Men (n =190)	0.95 (0.93–0.96)	<0.0001
Women (n = 453)	0.89 (0.86–0.90)	<0.0001
**Age (years)**		
30–49 (n = 91)	0.88 (0.83–0.92)	<0.0001
50–59 (n = 211)	0.83 (0.78–0.87)	<0.0001
60–70 (n = 341)	0.76 (0.71–0.80)	<0.0001

Supplementary Table S17. Prevalence of high or very high predicted 10-year cardiovascular disease risk among people with hypertension aged 30-70 without a history of cardiovascular disease in rural Thailand (the Thai cardiovascular risk score)

	Weighted prevalence (95% CI)	
	**Laboratory-based**	**Non-Laboratory based**
**Total**	33.4 (28.2–39)	50.9 (46.9–55.0)
**Sex**[k]		
Men	50.9 (37.1–64.6)[a†]	61.8 (58.0–65.4)[a†]
Women	30.4 (23.7–38)	57.1 (51.9–62.2)
**Age (years)**[l]		
30–49	4.2 (0.9–17.1)[c‡]	5.2 (1.4–17.3)[b†, c‡]
50–59	13.0 (6.4–24.6)[d*]	20.9 (12.9–32.1)[d‡]
60–70	52.4 (42.7–61.9)	84.2 (76.0–90.0)
**Geographical region**[m]		
Central	54.3 (44.2–64.0)[e‡, f‡, g‡]	73.2 (64.5–80.4)[e†, f‡, g†]
Northeast	39.6 (34.0–45.6)[h*, i†]	59.4 (55.6–63.1)[h‡]
North	27.5 (18.1–39.3)	45.4 (39.2–51.8)[j*]
South	26.2 (18.5–35.7)	57.1 (46.4–67.2)

*p<0.05, †p<0.01, ‡p<0.001

[a]Men vs Women

[b]30-49 vs 50-59, [c]30-49 vs 60-70, [d]50-59 vs 60-70

[e]Central vs Northeast, [f]Central vs North, [g]Central vs South, [h]Northeast vs North, [i]Northeast vs South, [j]North vs South

[k]age-adjusted, [l]sex-adjusted, [m]age- and sex-adjusted

Supplementary Table S18. Sex distribution of people with hypertension receiving care at 36 primary care units (PCUs) in rural Thailand*

	**Sex distribution**		**Total**
	**Men n (%)**	**Women n (%)**	
**Overall**	2,741 (35.2)	5,036 (64.8)	7,777
**Geographical Region**			
Central (8 PCUs)	585 (33.8)	1147 (66.2)	1,732
Northeast (12 PCUs)	624 (36.3)	1093 (63.7)	1,717
North (8 PCUs)	794 (37.8)	1309 (62.2)	2,103
South (8 PCUs)	738 (33.2)	1487 (66.8)	2,225

*People with a hypertension diagnosis (ICD-10: I10) receiving care at PCU before June 1, 2023, appeared on the list.

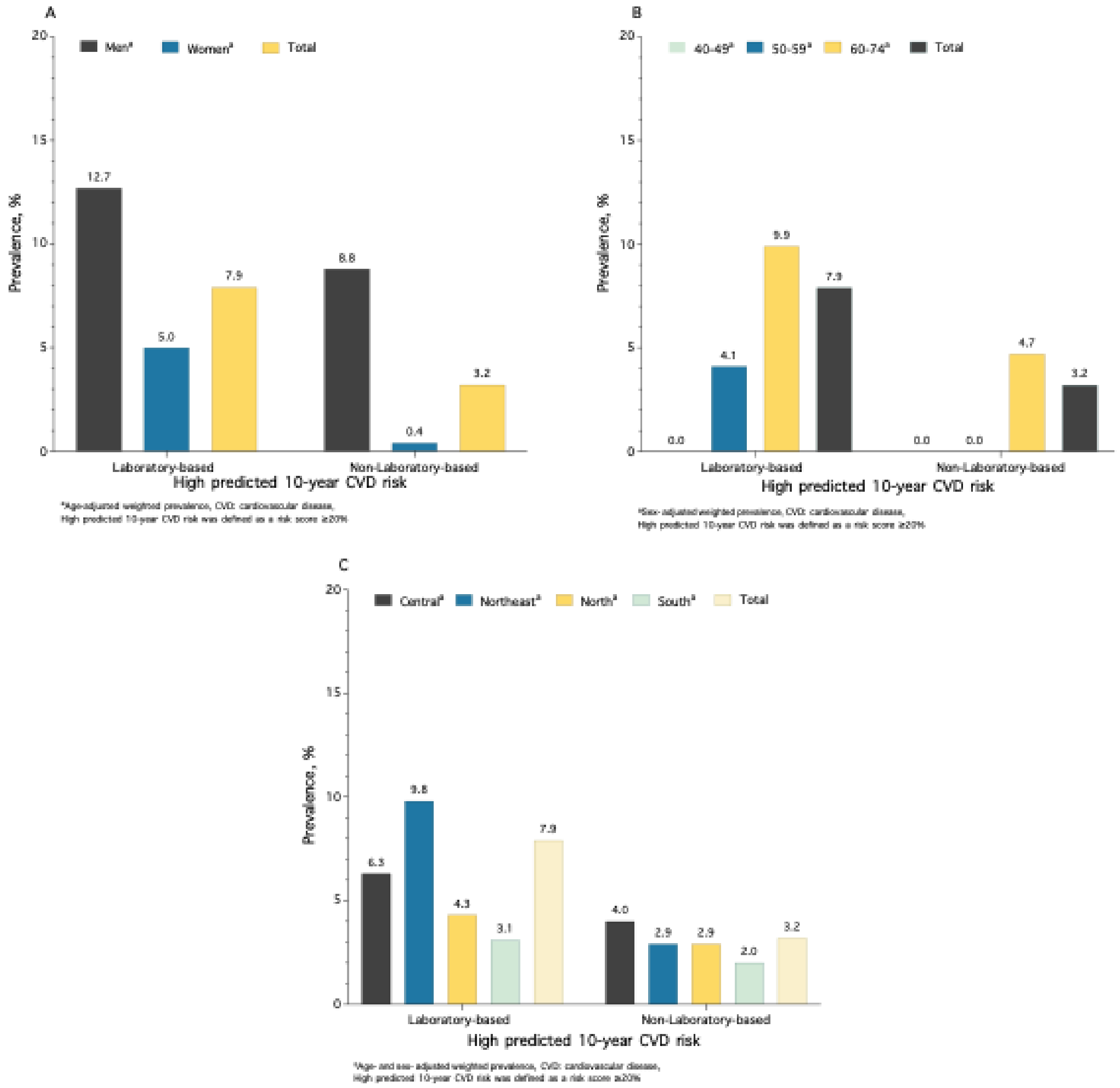

Supplementary Figure S2. Prevalence of high or very high predicted 10-year cardiovascular disease risk (2019 WHO) among people with hypertension aged 40-74 without a history of cardiovascular disease in rural Thailand, stratified by sex (A), age (B), and geographical region (C)

References of Supplemetary Methods

1. Sakboonyarat B, Rangsin R. Characteristics and clinical outcomes of people with hypertension receiving continuous care in Thailand: a cross-sectional study. *The Lancet Regional Health-Southeast Asia*. 2024;21.
2. Lambert EV, Steyn K, Stender S, Everage N, Fourie JM, Hill M. Cross-cultural validation of the hill-bone compliance to high blood pressure therapy scale in a South African, primary healthcare setting. *Ethn Dis*. 2006;16(1):286-291.
3. Kim MT, Hill MN, Bone LR, Levine DM. Development and testing of the hill-bone compliance to high blood pressure therapy scale. *Prog Cardiovasc Nurs*. 2000;15(3):90-96.
4. Wongpakaran N, Wongpakaran T. The Thai version of the PSS-10: An Investigation of its psychometric properties. *Biopsychosoc Med*. 2010;4:1-6.
5. Spitzer RL, Kroenke K, Williams JBW, Löwe B. A brief measure for assessing generalized anxiety disorder: the GAD-7. *Arch Intern Med*. 2006;166(10):1092-1097.
6. Lotrakul M, Sumrithe S, Saipanish R. Reliability and validity of the Thai version of the PHQ-9. *BMC Psychiatry*. 2008;8(1):1-7.
7. Kunanon S, Chattranukulchai P, Chotruangnapa C, et al. 2019 Thai Guidelines on the Treatment of Hypertension: Executive Summary. *JOURNAL OF THE MEDICAL ASSOCIATION OF THAILAND*. 2021;104(10):1729-1738.
8. Gabriel J, Anne LAM, Thiele ITI, Mae SC, Elizabeth PP. Impact of waist circumference measurement variation on the diagnosis of metabolic syndrome. *Philipp J Intern Med*. Published online 2010:7-17.
9. Loehr LR, Rosamond WD, Poole C, et al. Association of multiple anthropometrics of overweight and obesity with incident heart failure: the Atherosclerosis Risk in Communities study. *Circ Heart Fail*. 2009;2(1):18-24.
10. Jevon P. Procedure for recording a standard 12-lead electrocardiogram. *British Journal of Nursing*. 2010;19(10):649-651.
11. Nordestgaard BG, Langsted A, Mora S, et al. Fasting is not routinely required for determination of a lipid profile: Clinical and laboratory implications including flagging at desirable concentration cut-points - A joint consensus statement from the European Atherosclerosis Society and European Federation of Clinical Chemistry and Laboratory Medicine. *Eur Heart J*. 2016;37(25). doi:10.1093/eurheartj/ehw152
12. Sakboonyarat B, Rangsin R. Characteristics and clinical outcomes of people with hypertension receiving continuous care in Thailand: a cross-sectional study. *The Lancet Regional Health - Southeast Asia*. Published online 2023:100319. doi:https://doi.org/10.1016/j.lansea.2023.100319
13. World Health Organization. Standard STEPS instrument. October 1, 2020. Accessed March 18, 2024. https://www.who.int/publications/m/item/standard-steps-instrument
14. Craig CL, Marshall AL, Sjöström M, et al. International physical activity questionnaire: 12-Country reliability and validity. *Med Sci Sports Exerc*. 2003;35(8). doi:10.1249/01.MSS.0000078924.61453.FB

15. Brown WJ, Trost SG, Bauman A, Mummery K, Owen N. Test-retest reliability of four physical activity measures used in population surveys. *J Sci Med Sport*. 2004;7(2). doi:10.1016/S1440-2440(04)80010-0
16. National Center for Health Statistics. Adult Tobacco Use Information. August 29, 2017. Accessed March 18, 2024. https://www.cdc.gov/nchs/nhis/tobacco/tobacco_glossary.htm
17. National Center for Health Statistics. Glossary - Alcohol. March 6, 2018. Accessed March 18, 2024. https://www.cdc.gov/nchs/nhis/alcohol/alcohol_glossary.htm
18. WHO. ICD-10: International Statistical Classification of Diseases and Related Health Problems: Tenth Revision. Published online 2016. Accessed February 2, 2024. https://icd.who.int/browse10/2016/en#/I20-I25
19. Elsayed NA, Aleppo G, Aroda VR, et al. 2. Classification and Diagnosis of Diabetes: Standards of Care in Diabetes—2023. *Diabetes Care*. 2023;46. doi:10.2337/dc23-S002
20. Organization WH. The Asia-Pacific perspective: redefining obesity and its treatment. Published online 2000.
21. Organization WH. Waist circumference and waist-hip ratio: report of a WHO expert consultation, Geneva, 8-11 December 2008. Published online 2011.
22. Lloyd-Jones DM, Allen NB, Anderson CAM, et al. Life’s Essential 8: Updating and Enhancing the American Heart Association’s Construct of Cardiovascular Health: A Presidential Advisory from the American Heart Association. *Circulation*. 2022;146(5). doi:10.1161/CIR.0000000000001078
23. Mendis S, Thygesen K, Kuulasmaa K, et al. World Health Organization definition of myocardial infarction: 2008-09 revision. *Int J Epidemiol*. 2011;40(1). doi:10.1093/ije/dyq165
24. Prineas RJ, Crow RS, Zhang ZM. *The Minnesota Code Manual of Electrocardiographic Findings*. Springer Science & Business Media; 2009.
25. Yu Z, Song J, Cheng L, et al. Peguero-Lo Presti criteria for the diagnosis of left ventricular hypertrophy: A systematic review and meta-analysis. *PLoS One*. 2021;16(1 January). doi:10.1371/journal.pone.0246305
26. Kaptoge S, Pennells L, De Bacquer D, et al. World Health Organization cardiovascular disease risk charts: revised models to estimate risk in 21 global regions. *Lancet Glob Health*. 2019;7(10):e1332-e1345.
27. Kwon D, Yi JJ, Ohrr H, Yi SW. Total cholesterol and mortality from ischemic heart disease and overall cardiovascular disease in Korean adults. *Medicine (United States)*. 2019;98(36). doi:10.1097/MD.0000000000017013
28. Faculty of Medicine Ramathibodi Hospital MU. Thai CV risk score 2015. 2015. Accessed November 17, 2025. https://www.rama.mahidol.ac.th/cardio_vascular_risk/thai_cv_risk_score/tcvrs_en.html
29. Vathesatogkit P, Woodward M, Tanomsup S, et al. Cohort profile: the electricity generating authority of Thailand study. *Int J Epidemiol*. 2012;41(2):359-365.

STROBE Statement—checklist of items that should be included in reports of observational studies

Clinical Epidemiology of Hypertension in Rural Thailand: A Nationwide Cross-sectional Study

	Item No	Recommendation	Page No
**Title and abstract**	1	(*a*) Indicate the study's design with a commonly used term in the title or the abstract	1-2
		(*b*) Provide in the abstract an informative and balanced summary of what was done and what was found	2-3
**Introduction**			
Background/rationale	2	Explain the scientific background and rationale for the investigation being reported	5
Objectives	3	State specific objectives, including any prespecified hypotheses	6
**Methods**			
Study design	4	Present key elements of study design early in the paper	6
Setting	5	Describe the setting, locations, and relevant dates, including periods of recruitment, exposure, follow-up, and data collection	6, Supplementary materials
Participants	6	(*a*) *Cohort study*—Give the eligibility criteria, and the sources and methods of selection of participants. Describe methods of follow-up<br>*Case-control study*—Give the eligibility criteria, and the sources and methods of case ascertainment and control selection. Give the rationale for the choice of cases and controls<br>*Cross-sectional study*—Give the eligibility criteria, and the sources and methods of selection of participants	6-7, Supplementary materials
		(*b*) *Cohort study*—For matched studies, give matching criteria and number of exposed and unexposed<br>*Case-control study*—For matched studies, give matching criteria and the number of controls per case	N/A
Variables	7	Clearly define all outcomes, exposures, predictors, potential confounders, and effect modifiers. Give diagnostic criteria, if applicable	8-10, Supplementary materials
Data sources/ measurement	8*	For each variable of interest, give sources of data and details of methods of assessment (measurement). Describe comparability of assessment methods if there is more than one group	*7-8*, Supplementary materials
Bias	9	Describe any efforts to address potential sources of bias	7-8
Study size	10	Explain how the study size was arrived at	7, Supplementary materials
Quantitative variables	11	Explain how quantitative variables were handled in the analyses. If applicable, describe which groupings were chosen and why	8-10, Supplementary materials
Statistical methods	12	(*a*) Describe all statistical methods, including those used to control for confounding	10-11

		(*b*) Describe any methods used to examine subgroups and interactions	10-11
		(*c*) Explain how missing data were addressed	N/A
		(*d*) *Cohort study*—If applicable, explain how loss to follow-up was addressed<br>*Case-control study*—If applicable, explain how matching of cases and controls was addressed<br>*Cross-sectional study*—If applicable, describe analytical methods taking account of sampling strategy	10-11
		(*e*) Describe any sensitivity analyses	N/A
**Results**			
Participants	13*	(a) Report numbers of individuals at each stage of study—eg numbers potentially eligible, examined for eligibility, confirmed eligible, included in the study, completing follow-up, and analysed	11-12, Table 1
		(b) Give reasons for non-participation at each stage	11
		(c) Consider use of a flow diagram	n/a
Descriptive data	14*	(a) Give characteristics of study participants (eg demographic, clinical, social) and information on exposures and potential confounders	11
		(b) Indicate number of participants with missing data for each variable of interest	33-34
		(c) *Cohort study*—Summarise follow-up time (eg, average and total amount)	n/a
Outcome data	15*	*Cohort study*—Report numbers of outcome events or summary measures over time	n/a
		Case-control study—Report numbers in each exposure category, or summary measures of exposure	n/a
		Cross-sectional study—Report numbers of outcome events or summary measures	12-16, 33-37, Supplementary materials
Main results	16	(*a*) Give unadjusted estimates and, if applicable, confounder-adjusted estimates and their precision (eg, 95% confidence interval). Make clear which confounders were adjusted for and why they were included	12-16, 33-37, Supplementary materials
		(*b*) Report category boundaries when continuous variables were categorized	12-16, 33-37, Supplementary materials
		(*c*) If relevant, consider translating estimates of relative risk into absolute risk for a meaningful time period	n/a
Other analyses	17	Report other analyses done—eg analyses of subgroups and interactions, and sensitivity analyses	n/a
**Discussion**			
Key results	18	Summarise key results with reference to study objectives	16
Limitations	19	Discuss limitations of the study, taking into account sources of potential bias or imprecision. Discuss both direction and magnitude of any potential bias	22-23

Interpretation	20	Give a cautious overall interpretation of results considering objectives, limitations, multiplicity of analyses, results from similar studies, and other relevant evidence	16-22
Generalisability	21	Discuss the generalisability (external validity) of the study results	23
**Other information**			
Funding	22	Give the source of funding and the role of the funders for the present study and, if applicable, for the original study on which the present article is based	24

*Give information separately for cases and controls in case-control studies and, if applicable, for exposed and unexposed groups in cohort and cross-sectional studies.

Note: An Explanation and Elaboration article discusses each checklist item and gives methodological background and published examples of transparent reporting. The STROBE checklist is best used in conjunction with this article (freely available on the Web sites of PLoS Medicine at http://www.plosmedicine.org/, Annals of Internal Medicine at http://www.annals.org/, and Epidemiology at http://www.epidem.com/). Information on the STROBE Initiative is available at www.strobe-statement.org.
